# Prevalence of Shiga toxin-producing *Escherichia coli, Salmonella*, and *Campylobacter* species among diarrheal patients from three major hospitals in Ethiopia

**DOI:** 10.1371/journal.pgph.0004407

**Published:** 2025-04-21

**Authors:** Amete Mihret Teshale, Woldaregay Erku Abegaz, Binyam Moges Azmeraye, Desalegne Degefaw, Devin LaPolt, Zelalem Bonger, Alem Abrha Kalayu, Eyasu Tigabu, Lina Gazu, Getnet Yimer, Ebba Abate, Estifanos Tsige, Geremew Tasew, Yadeta Dessie, Gashaw Biks, James A. Barkley, Ariel V. Garsow, Aaron Beckiewicz, Silvia Alonso, Barbara Kowalcyk

**Affiliations:** 1 National Clinical Bacteriology and Mycology Reference Laboratory, Ethiopian Public Health Institute, Addis Ababa, Ethiopia; 2 Department of Microbiology, Immunology and Parasitology, College of Health Sciences, Addis Ababa University, Addis Ababa, Ethiopia; 3 The Ohio State University Global One Health Initiative, Addis Ababa, Ethiopia; 4 Department of Food Science and Technology, Center for Foodborne Illness Research and Prevention, The Ohio State University, Columbus, Ohio, United States of America; 5 Animal and Human Health Program, International Livestock Research Institute, Addis Ababa, Ethiopia; 6 Department of Genetics and Penn Center for Global Genomics and Health Equity, Perelman School of Medicine University of Pennsylvania, Philadelphia, United States of America; 7 Project HOPE Ethiopia, Addis Ababa, Ethiopia; 8 School of Public Health, College of Health and Medical Sciences, Haramaya University, Harar, Ethiopia; 9 African Population and Health Research Center, Nairobi, Kenya; 10 Institute of Public Health, College of Medicine and Health Sciences, University of Gondar, Gondar, Ethiopia; 11 Translational Data Analytics Institute, The Ohio State University, Columbus, Ohio, United States of America; 12 Milken Institute School of Public Health, The George Washington University, Washington, DC, United States of America; University of Oxford, UNITED KINGDOM OF GREAT BRITAIN AND NORTHERN IRELAND

## Abstract

Diarrheal illness remains a major global health challenge, causing millions of deaths annually. Non-typhoidal *Salmonella* (NTS), Shiga toxin-producing *Escherichia coli* (STEC), and *Campylobacter* species (CAMPY) significantly contribute to this burden. Given the limited information on these pathogens in Ethiopia, this study aimed to estimate their prevalence among diarrhea patients in Ethiopia and identify risk factors for infection. A cross-sectional study was conducted from October 2021 to November 2022 in three hospitals in Ethiopia (Addis Ababa, Gondar, and Harar). Sociodemographic characteristics, clinical signs and symptoms were collected from study participants using a structured questionnaire. Stool samples were tested for NTS, STEC, and CAMPY using standardized methods. The prevalence of targeted pathogens was estimated overall and by study sites. Univariable and multivariable logistic regression were used to identify associated factors. A total of 2,331 patients were enrolled. The overall prevalence of NTS, STEC (*stx* only), STEC (*stx* + *eae*), and CAMPY was 1.29% (95%CI: 0.91, 1.84), 12.56% (95%CI: 11.29, 13.98), 3.43% (95%CI: 2.77, 4.25), and 4.46% (95%CI: 4.61, 8.00), respectively. Harar had the highest prevalence of all the pathogens compared to Addis Ababa and Gondar. Odds of NTS in Harar were over 10 (AOR: 10.43: 95%CI: 2.95, 69.20) and 3.5 times (AOR: 3.57: 95%CI: 1.50, 9.90) higher than that in Addis Ababa and Gondar, respectively. Odds of STEC (*stx* only) in the dry (AOR: 1.97: 95%CI: 1.37, 2.90) and long rainy (AOR: 1.80: 95%CI: 1.20, 2.69) seasons were nearly twice the odds in the short rainy season. Odds of CAMPY infection decreased by 3.29% (AOR: 0.97: 95%CI: 0.95, 0.98) with every one-year increase in age. Moreover, the odds of CAMPY infection for rural residents (AOR: 1.93, 95%CI: 1.15, 3.19) were nearly twice that of urban residents. This is the first study to estimate the prevalence of NTS, STEC, and CAMPY simultaneously across all age groups and diverse regions in Ethiopia, revealing significant variations. Results can be used to understand the burden of disease, inform clinical management and risk mitigation strategies to reduce illness in Ethiopia.

## Author summary

Diarrheal diseases caused by foodborne bacterial pathogens such as non-typhoidal *Salmonella* (NTS), Shige toxin-producing *Escherichia coli* (STEC), and *Campylobacter* spp (CAMPY) are major public health concerns worldwide. In Ethiopia, limited data exist on the number of illnesses associated with these pathogens, which makes it challenging to implement targeted prevention strategies. A cross-sectional survey was conducted to fill this gap by estimating the prevalence of these pathogens among diarrheal patients submitting stool samples to three clinical laboratories. A total of 2331 diarrheal patients were recruited in the study from the three major cities of Ethiopia. The overall prevalence of NTS, STEC (*stx* only), STEC (*stx* + *eae*), and CAMPY was 1.29% (95%CI: 0.91, 1.84), 12.56% (95%CI: 11.29, 13.98), 3.43% (95%CI: 2.77, 4.25), and 4.46% (95%CI: 4.61, 8.00), respectively. The study found varying prevalence rates, with Harar showing the highest prevalence for all pathogens. Seasonal differences were also noted, such as higher odds of STEC infections in the dry and long rainy seasons. Additionally, rural residents were more likely to be infected with CAMPY than urban residents. These findings provide important insights into the causes of diarrheal disease in Ethiopia, guiding public health interventions and providing a foundation for more effective prevention, control, and clinical management strategies to reduce the impact of diarrheal diseases in the country.

## Introduction

Diarrhea, a common symptom of foodborne disease (FBD), is a significant public health concern worldwide. Globally, diarrhea is the third leading contributor to disability-adjusted life years (DALYs) among children under 10 years of age [1] and the eighth leading cause of death among all ages [2]. Low-and middle-income countries (LMICs) are disproportionately impacted [3], with the highest burden being found in Africa, South Asia, and Southeast Asia [4].

Infectious agents such as Non-typhoidal *Salmonella* (NTS), Shiga toxin-producing *Escherichia coli* (STEC), and *Campylobacter* spp. (CAMPY) present significant global health risks, causing severe diarrheal illness and potential long-term complications. These pathogens are commonly transmitted through consumption of contaminated food and water, contact with animals, or person-to-person exposure [5]. Among the common causes of foodborne outbreaks, NTS is prevalent globally and can lead to severe gastroenteritis [6]. While less frequent, STEC infections can cause life-threatening complications such as hemolytic uremic syndrome (HUS), particularly among vulnerable populations such as the elderly and young children [7]. Severe STEC infections and complications are associated with strains that have a combination of *stx* (*stx1*/*stx2*) and *eae* genes [8,9]. Infections caused by CAMPY carry significant health and economic burdens, causing millions of illnesses annually and the potential for long-term complications such as Guillain-Barré Syndrome [10].

According to the World Health Organization, the disease burden of these pathogens in Ethiopia is significant [11]. For example, NTS causes an estimated 932,000 illnesses and 1,340 deaths annually in Ethiopia, resulting in 101,000 DALYs. Similarly, CAMPY causes an estimated 2,380,000 illnesses and 671 deaths annually, resulting in 61,500 DALYs. The reported burden of STEC in Ethiopia is estimated to be 10-fold lower than the global burden, although this is likely due to limited data on the burden of STEC in Africa. Notably, these estimates were derived from data obtained from WHO and updated using data from the Global Burden of Disease study.

Despite the high burden associated with these pathogens, research on the affected populations and etiologies underlying NTS, STEC, and CAMPY across different geographic regions in Ethiopia is limited. Evidence on diarrheal illness in Ethiopia has predominantly focused on specific age groups and often overlooked the broader landscape of foodborne pathogens [12–17]. Most research studies have focused on one single pathogen, such as CAMPY [18], *Salmonella spp*. [19], or *E. coli* O157: H7 [20], but none have considered the burden of multiple pathogens, particularly NTS, STEC, and CAMPY, simultaneously on the overall population. Therefore, the objective of this study was to estimate the prevalence of NTS, STEC, and CAMPY among diarrheal patients in Ethiopia, identify factors associated with infection, and provide evidence on the burden of these pathogens for future targeted interventions to mitigate public health risks.

## Methods

### Ethics statement

Ethical approvals were obtained from the Ohio State University (Protocol 2020H0266); International Livestock Research Institute (Protocol IREC2020-47 and amendment IREC2020-47.1); Ethiopian Public Health Institute (Protocol EPHI-IRB-311-2020); Addis Ababa University College of Health Sciences Institutional Review Board (Protocol 075/21/DMIP); Yekatit-12 Hospital Medical College (Protocol 68/21); University of Gondar (Protocol V/P/RCS/05/101/2020); Haramaya University (Protocol IHRERC/020/2021); and George Washington University (Protocol NCR235278).

Additional information regarding the ethical, cultural, and scientific considerations specific to inclusivity in global research is included in te Supporting Information (S1 Text).

A cross-sectional study was conducted from 11 October 2021 to 30 November 2022 to estimate the prevalence of NTS, STEC and CAMPY in diarrheal patients submitting physician-requested stool samples to the clinical laboratories at three hospitals in Ethiopia: 1) Yekatiti-12 Hospital, a specialized teaching hospital in Addis Ababa; 2) University of Gondar Comprehensive Specialized Hospital, a referral and teaching hospital in Gondar; and 3) Hiwot Fana Comprehensive Specialized Hospital, a teaching hospital in Harar. These study sites were selected because they serve a large and diverse population and are representative of the three predominant ecosystems in Ethiopia: central (Addis Ababa), north (Gondar), and south-eastern (Harar).

**Fig 1:**
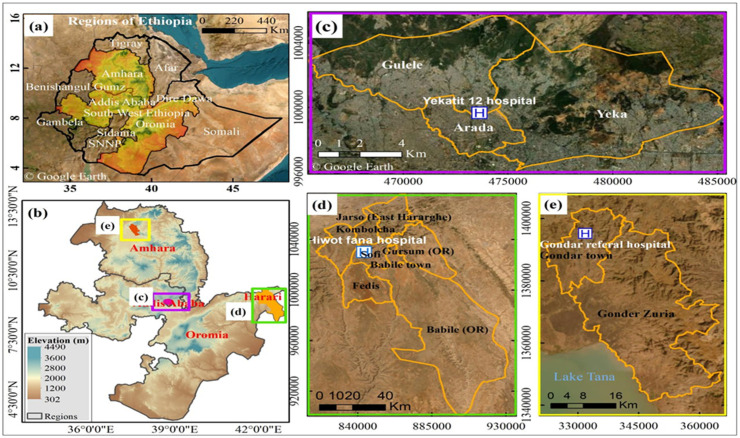
Study areas of sample collection in Ethiopia, eastern Africa **(a)**, from the three target regions **(b)**, and the three hospitals, Yekatit 12 Hospital **(c)**, Hiwot Fana Hospital **(d)**, and University of Gondar Comprehensive Specialized Hospital **(e), (source of the administrative boundaries from central statistics agency of Ethiopia; background google source data: Esri, Maxar, GeoEye, Earthstar Geographics, CNES/Airbus DS, USDA, USGS, AeroGRID, IGN, and the GIS User Community), (mapped by Mohammed Ahmed).**

Patients were eligible to participate in the study if they 1) were diagnosed with diarrhea by their physician; 2) were ordered by their physician to provide a stool sample for testing; 3) had not taken antibiotics in the last two weeks; and 4) lived in the defined catchment area for the hospital. The catchment areas for the three hospitals were defined using information from the local health bureaus in relation to where individuals typically seek medical care (Fig 1). The catchment area for Yekatit-12 Hospital was defined to be the sub-cities of Gulele, Yeka, and Arada. The catchment area for the University of Gondar Comprehensive Specialized Hospital was defined to be Gondar town and surrounding districts woredas (Sabiya Sabina, Diba Defecha, Fenter, Teda town, Azezo Teklehaimanot, Mariam Debir Deresgie, Loza Mariam, Killil Rufaelna ena killil Eysesus, Dabrka, Aba intones, and Anchew Michael). The catchment area for Hiwot Fana Comprehensive Specialized University Hospital was defined to be the town of Harar and the adjacent district areas of East Hararge Zone of Oromia, including Jarso, Gursum, Kombolcha, Maya (the former name Haramaya), Fedis and Babile.

Eligible patients were recruited to participate in the study when they submitting stool samples to the hospitals’ clinical laboratories, and written informed consent was obtained. Parental consent was obtained for children under the age of 18, and assent was also obtained from children aged 12–17 years of age. Following consent, participants completed a pre-tested survey via an interview with a trained study team member. The survey included questions about socio-demographic characteristics (e.g., age, gender, marital status, education level, occupation/source of income); the current diarrheal illness (e.g., symptoms, days of illness, treatment); costs related to the diarrheal illness; health-seeking behaviors; environmental exposures (e.g., animal, water); household food purchasing, handling, and consumption practices; and knowledge of foodborne illness and food safety (S2 Text).

Stool samples were obtained according to physician instructions and tested for NTS, STEC, and CAMPY using standardized protocols that included bacterial culture, biochemical tests, immunological assay, and polymerase chain reaction (PCR). Submitted stool samples were screened and excluded if the sample was unlabeled or mislabeled, received in a non-sterile stool collection cup, or leaking container. Samples collected at a location other than the microbiology laboratory (e.g., parasitology laboratory) were transferred to Cary-Blair transport media and transported to the testing microbiology laboratories using a cold-chain transportation system. Samples with qPCR threshold cycle (Ct) values <37 were considered positive, while samples with Ct values >40 were considered negative.

For NTS isolation and identification, portions of stool samples were placed into Selenite-F broth using a 10µl plastic inoculating loop and incubated at 35 ± 2 °C overnight [21]. Samples from the enriched broth were plated onto MacConkey (MAC, Difco, USA) and Xylose-Lysine-Deoxycholate (XLD, Difco, USA) agar plates, and then incubated aerobically at 35 ± 2 °C overnight. Colonies were examined for NTS characteristics, including hydrogen-sulfide production on XLD and non-lactose fermentation on MAC. Biochemical analyses were conducted using Triple Sugar Iron Agar (TSIA, Difco, USA); Lysine Iron Agar (LIA, Difco, USA); Simmon Citrate Agar (SCA, Difco, USA); Urease Agar (UA, Difco, USA); and Sulfide Indole Motility Agar (SIMA, Difco, USA). Identification was based on standard biochemical characteristics. Molecular confirmation was conducted targeting the *invA* gene using MIC qPCR [22] (S3 Text). DNA was extracted from a pure colony using the boiling method [23]. Briefly, colonies were suspended in sterile nuclease-free water (250 µl) and centrifuged at 14,000 rpm for 10 minutes, with the resulting supernatant being discarded. Pellets were resuspended in 250 µl nuclease-free water, boiled at 100 °C for 10 minutes, cooled for 15 minutes, and centrifuged at 14,000 rpm for 10 minutes. The supernatant was used as DNA templates. The amount and purity of the DNA template were measured using a NanoDrop apparatus [24]. Monoplex PCR was conducted according to a previously used protocol [25] and included an initial denaturation at 95 °C for 2 minutes, followed by 50 cycles at 95 °C for 10 seconds and 60 °C for 60 seconds. *Salmonella* Enteritidis (ATCC 14028) served as the positive control, with nuclease-free water serving as the negative control. The positive and negative results were interpreted based on the Ct value indicated above.

For STEC isolation and identification, portions of stool samples were plated onto ChromSTEC agar (ChromSTEC, France) using a 10µl plastic inoculating loop and incubated aerobically at 35±2 °C overnight. Mauve colonies were assumed to be indicative of STEC [26]. Molecular confirmation targeting *stx1*/*stx2* and *eae* genes was conducted using MIC qPCR was conducted (S3 Text). DNA was extracted from a pure colony using the boiling method [23], as described above. The supernatant was used as DNA templates, and the amount and purity of DNA were measured using a NanoDrop apparatus [24]. The assay followed ISO/TS 13136 guidelines [27], with a 20µl reaction volume containing primer pairs, dual, fluorescent-labeled probes, and master mix, with 1 µl of DNA template. Multiplex qPCR was used in this case where the following steps were followed: pre-amplification at 95 °C for 2 minutes, followed by 50 cycles of denaturation at 95 °C for 10 seconds, and annealing/extension at 60 °C for 10 seconds. *E. coli* O157 (ATCC 35150) serotype served as the positive control, with nuclease-free water serving as the negative control. Stool samples positive for *stx1/stx2* or both were considered to be STEC (*stx* only) positive. Stool samples positive for *stx1/stx2* and *eae* were considered to be STEC (*stx* + *eae*) positive, a subset of STEC that is associated with severe illness and complications [9,28]. The two definitions are set because severe STEC infections and complications are associated with strains that have a combination of *stx* (stx1/stx2) and *eae* genes [8,9]. The positive and negative results were interpreted using the Ct value indicated above.

For CAMPY detection, stool samples were tested using an immunoassay technique (Campy Quick Check, USA) [29]. Diluent (750 µL) was added to the test tube; one drop of conjugate was added to each tube containing the diluent, and the stool specimen (25 µL) was added into the tube of diluent-conjugate. The diluted specimens were vortexed for 5–20 seconds and incubated for 30 minutes at room temperature. The diluent-conjugate-stool mixture (500 µL) was then transferred into the sample well of the membrane device and incubated for 15 minutes at room temperature. Wash buffer (300 µL) and two drops of substrate were added before examining the test result. Molecular confirmation of CAMPY was conducted by targeting CAMPY-specific *16S rRNA* genes using the MIC qPCR (S3 Text). DNA was extracted from frozen stool samples using the Fast Stool DNA Kit (Qiagen GmbH, Hilden, Germany) following the manufacturer’s protocol [30]. Monoplex qPCR was used with initial denaturation at 95 °C for 10 minutes, followed by 50 cycles of 95 °C for 15 seconds and 55 °C for 60 seconds. The 20µl reaction volume comprised primer pairs (1 µl each), probe (1 µl), master mix (12.5 µl), and nuclease-free water (0.5 µl). *C. jejuni* (ATCC 29411) and *C. coli* (ATCC 43478) served as the positive controls, with nuclease-free water served as the negative control. The positive and negative results were interpreted based on the Ct value indicated above.

Data were collected electronically using REDCap (National Center for Advancing Translational Sciences, Grant UL1TR001070) [31] and analyzed using R (R Core Team (2021) [32]). Descriptive statistics were used to summarize socio-demographic characteristics, symptoms, and laboratory test results. The prevalence of NTS, STEC, and CAMPY infection was calculated by dividing the number of positive stool samples for the targeted bacterial pathogens by the total number of stool samples tested. The respective 95% Wald confidence intervals were calculated using the binomial proportion test and logistic regression. Seasonal trends of NTS, STEC, and CAMPY were assessed overall by month and by study site using univariable and multivariable logistic regression. The adequacy of the models was assessed using goodness-of-fit tests, including the Akaike Information Criterion (AIC). Risk factors for a positive laboratory test result were identified for each pathogen and study site separately using univariable and multivariable logistic regression. The potential risk factors considered were gender, age, residence urbanicity (i.e., urban vs rural), and season. Initially, the season was classified as rainy or dry, but due to changing weather patterns, it was re-classified in the analysis as dry (October to February), short rain (March to May), and long rain (June to September) [33]. Risk factors significant at an alpha of 0.20 in the univariable model were included in the multivariable logistic regression model. The Bonferroni correction was used to adjust for multiple pairwise comparisons, but results were similar, and only uncorrected results were presented.

The planned sample size for this study was 2,304, with 768 patients being recruited from each region. This sample size provides 80% power to detect a difference in proportions of 0.05 between seasons and 0.16 between the three study sites at an alpha level of 0.05. Since data on the expected proportions of patients who would test positive for NTS, STEC, and CAMPY were unavailable, it was assumed, for sample size calculations, that 50% of individuals submitting specimens would test positive.

## Results

**Fig 2:**
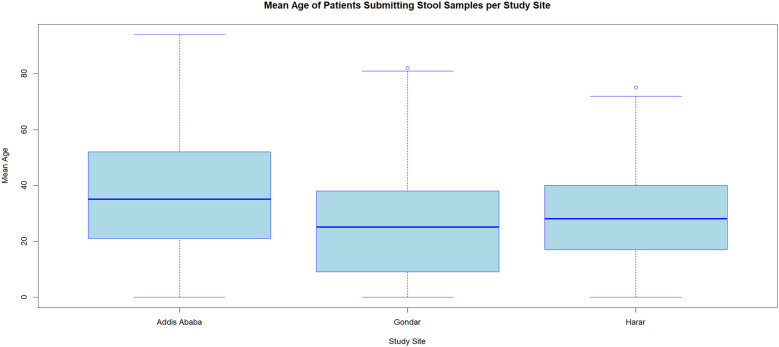
Mean age of patients submitting stool samples per study site.

A total of 2331 patients were enrolled in the study (Table 1), with a median age of 28 years (range: 2 months to 94 years) (Fig 2). All participants from Addis Ababa were urban residents, while about a quarter of participants from Harar and Gondar reported living in rural areas. Most participants in all study sites were male. Monthly household income varied by study site, with incomes being relatively lower in Addis Ababa (<2000 birr, and 2000 birr – 4000 birr) compared to Gondar (8000 birr – 10,000 birr) and Harar (6000 birr – 8000 birr). About half of the study participants were enrolled during the dry season. Abdominal pain and abdominal cramps were the most reported symptoms, and watery stool was the most common type of stool reported.

**Table 1:** Socio-demographic and clinical characteristics of participants by study site.

Characteristics	Categories	Addis Ababa	Gondar	Harar
		n (%)	n (%)	n (%)
Total patients		792 (33.98%)	767 (32.90%)	772 (33.12%)
Sex	Male	410 (51.80%)	432 (56.30%)	435 (56.30%)
	Female	382 (48.20%)	335 (43.70%)	337 (43.70%)
Residence Urbanicity	Urban	792 (100.00%)	596 (77.70%)	573 (74.20%)
	Rural	0 (0.00%)	171 (22.30%)	199 (25.80%)
Monthly Household income (Ethiopian birr)	<2000	203 (25.60%)	109 (14.20%)	30 (3.90%)
	2000–4000	195 (24.60%)	49 (6.40%)	108 (14.00%)
	4000–6000	124 (15.70%)	54 (7.00%)	116 (15.00%)
	6000–8000	114 (14.40%)	89 (11.60%)	200 (25.90%)
	8000–10,000	47 (5.90%)	204 (26.60%)	157 (20.30%)
	≥10,000	20 (2.50%)	114 (14.90%)	32 (4.10%)
	Unknown	89 (11.20%)	148 (19.30%)	129 (16.70%)
Season	Dry	435 (54.92%)	365 (47.59%)	341 (44.17%)
	Short Rains	127 (16.04%)	257 (33.51%)	142 (18.39%)
	Long Rains	230 (29.04%)	145 (18.90%)	289 (37.44%)
Types of stools^*^	Watery stool	370 (46.70%)	628 (81.90%)	624 (80.80%)
	Bloody stool	30 (12.60%)	197 (25.70%)	60 (7.80%)
	Mucoid stool	61 (7.70%)	269 (35.10%)	100 (12.90%)
Signs and symptoms^*^	Abdominal cramps	352 (44.40%)	535 (69.70%)	470 (60.90%)
	Undefined Abdominal pain	426 (53.80%)	541 (70.50%)	567 (73.40%)
	Fever	166 (20.90%)	345 (45.00%)	301 (38.90%)
	Nausea	218 (27.50%)	66 (8.60%)	106 (13.70%)
	Vomiting	122 (15.40%)	141 (18.40%)	181 (23.40%)
	Bloating	178 (22.50%)	41 (5.30%)	7 (0.90%)
	None	11 (1.39%)	9 (1.17%)	8 (1.04%)

*Patients could have multiple types of stools and/or symptoms. ETB: Ethiopian birr.

**Fig 3:**
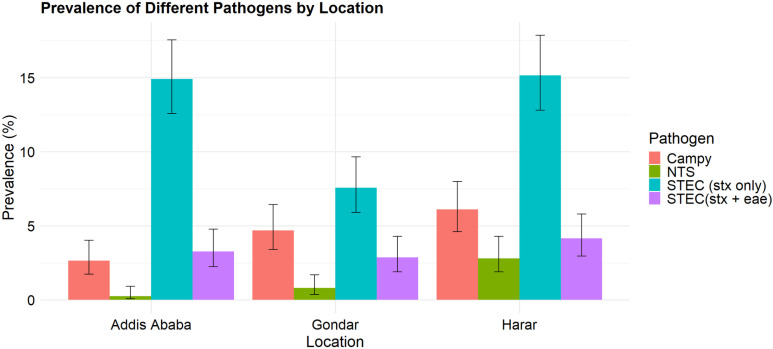
Prevaelnce of different pathogens by location.

Of the 2331 stool samples tested, 31.40% were presumptive positive for at least one pathogen; 69.26% of presumptive positives were confirmed using qPCR (Table 2). The overall prevalence of confirmed NTS, STEC (*stx* only), STEC (*stx + eae*), and CAMPY was 1.29% (95% CI: 0.90, 1.83); 12.56% (95% CI: 11.29, 13.98); 3.43% (95% CI: 2.77, 4.25); and 4.46% (95% CI: 3.70, 5.38), respectively (S1 Table). Prevalence varied by study site, with Harar having the highest prevalence for all pathogens (Fig 3). Significant disparities in pathogen prevalence between urban and rural residents were noted. For example, in urban areas, NTS was detected in 1.07% (95% CI: 0.70, 1.63), STEC (*stx* only) in 13.00% (95% CI: 11.59, 14.56), STEC (*stx + eae*) in 3.62% (95% CI: 2.88, 4.54), and CAMPY in 4.03% (95% CI: 3.24, 4.99). However, in rural areas, the prevalence was higher for NTS and CAMPY and lower for STEC (*stx* only) and STEC (*stx* + *eae*) (S1 Table).

**Table 2:** Positive stool samples by pathogen and study sites.

Pathogen		Study sites			
		Addis Ababa n (%)	Gondar n (%)	Harar n (%)	Total n (%)
Stool samples tested		792 (100%)	767 (100%)	772 (100%)	2,331 (100%)
NTS	Presumptive^*^	3 (0.38%)	8 (1.04%)	22 (2.80%)	33 (1.42%)
	Confirmed^**^	2 (0.30%)	6 (0.80%)	22 (2.80%)	30 (1.29%)
STEC (*stx* only)	Presumptive^*^	120 (14.15%)	62 (8.08%)	119 (15.41%)	301 (12.91%)
	Confirmed^**^	118 (14.90%)	58 (7.56%)	117 (15.15%)	293 (12.57%)
STEC (*stx* + *eae*)	Presumptive^*^	118 (14.90%)	58 (7.56%)	117 (15.15%)	293 (12.57%)
	Confirmed^**^	26 (3.28%)	22 (2.87%)	32 (4.15%)	80 (3.43%)
CAMPY	Presumptive^*^	21 (2.65%)	36 (4.69%)	48 (6.22%)	105 (4.50%)
	Confirmed^**^	21 (2.65%)	36 (4.69%)	47 (6.10%)	104 (4.46%)

*Bacteria suspected to be the targeted pathogens (NTS, STEC (*stx* only), STEC (*stx* + *eae*), or CAMPY).

**Bacteria identified or detected as targeted pathogens (NTS, STEC (*stx* only), STEC (*stx* + *eae*), CAMPY) after PCR confirmation.

**Fig 4:**
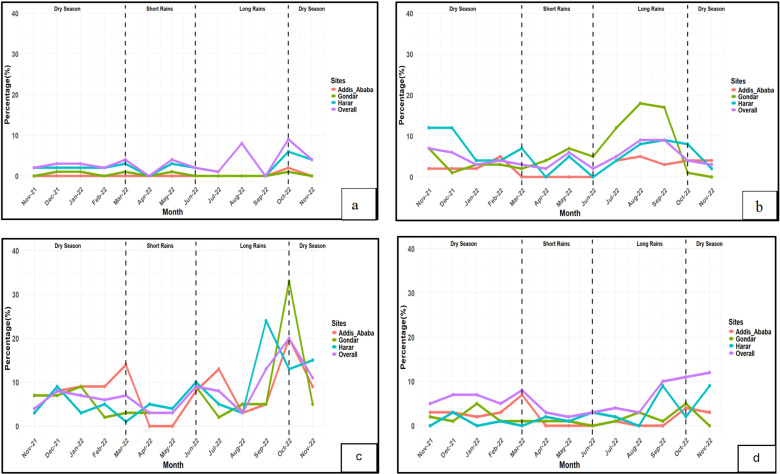
(a) Seasonal trends of non-typhoidal *Salmonella* by study sites. (b) Seasonal trends of *Campylobacter species* by study sites. (c) Seasonal trends of Shiga toxin-producing *Escherichia coli* by study sites. (d) Seasonal trends of Shiga toxin-producing *Escherichia coli* (*stx* + *eae*) by study sites.

The seasonal trends in the prevalence of NTS, STEC (*stx* only), STEC (*stx* + *eae*), and CAMPY varied by study site and by pathogen (Fig 4). For NTS, prevalence peaked during the long rains in August 2022 and the dry season in October 2022, with October showing the highest proportion across all study sites (Fig 4a). STEC (*stx* only) prevalence fluctuated significantly, with peaks in the long rains (August and September) and the dry season (October). Gondar had the highest peak in October, followed by Harar in September (Fig 4c). STEC (*stx* + *eae*) prevalence peaked during the dry season and declined during the rainy seasons, with noticeable peaks in Harar in September and November 2022 (Fig 4d). CAMPY prevalence had significant seasonal and site-specific variations, with peaks in August and September. Gondar showed a significant peak during the long rainy season, particularly in August 2022, while Harar peaked in November and December 2021, and September 2022. Addis Ababa remained relatively stable, with slight fluctuations between seasons and no reports during the short rainy seasons (Fig 4b).

**Fig 5:**
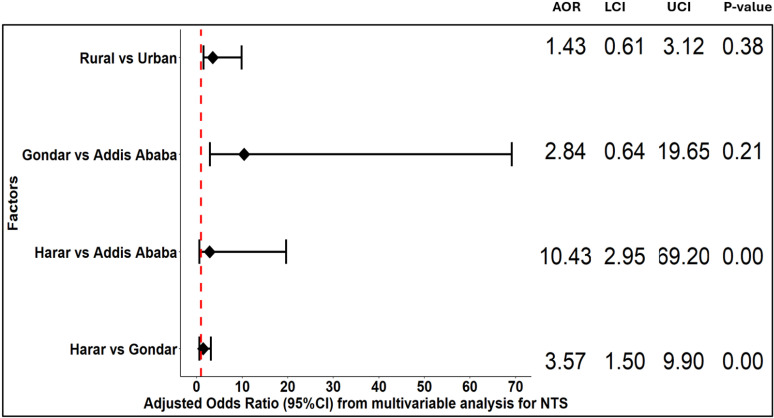
Odds ratio of multivariable analysis of factors for non-typhoidal *Salmonella.* **AOR**: Adjusted odds ratio, **LCI**: Wald lower confidence interval, **UCI**: Wald upper confidence interval.

NTS infection was associated with study sites and residence urbanicity (Fig 5). Odds of NTS infection were significantly higher in Harar compared to Addis Ababa and Gondar in the univariable analysis, but these associations were not significant in the multivariable analysis (S2 Table). Odds of NTS infection were also significantly higher in rural residents compared to urban residents in the univariable analysis, but this association was not significant in the multivariable analysis. Age, sex, and season were not significantly associated with NTS infection.

**Fig 6:**
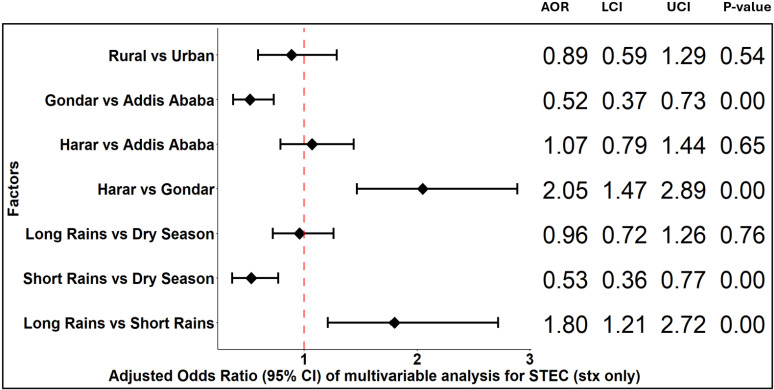
Odds ratio of multivariable analysis of factors for Shiga toxin-producing *Escherichia coli* (*stx* only). **AOR**: Adjusted odds ratio, **LCI**: Wald lower confidence interval, **UCI**: Wald upper confidence interval.

**Fig 7:**
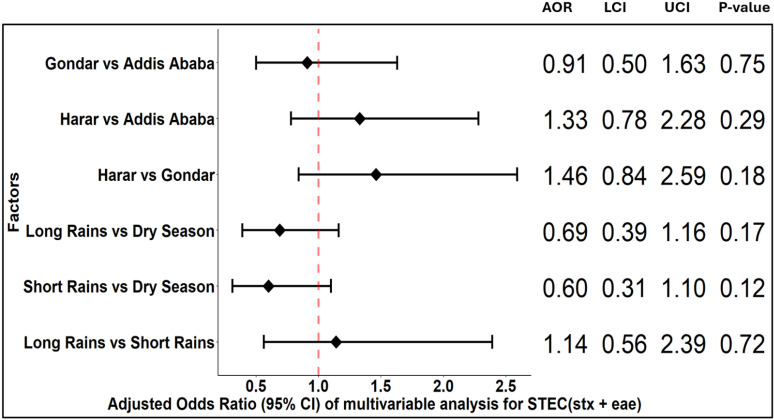
Odds ratio of multivariable analysis of factors for Shiga-toxin producing *Escherichia coli* (*stx* **+** ***eae*).** **AOR**: Adjusted odds ratio, **LCI**: Wald lower confidence interval, **UCI**: Wald upper confidence interval.

STEC (*stx* only) infection was associated with study site and season (Fig 6). The odds of STEC (*stx* only) infection were significantly higher in Harar and Addis Ababa compared to Gondar in the univariable and multivariable analyses. Odds of STEC (*stx* only) infection were also significantly higher in the dry season and long rain seasons compared to the short rain season in both the univariable and multivariable analyses (S2 Table). The odds of STEC (*stx* only) infection were marginally higher in rural residents compared to urban residents in the univariable analysis, but this association was not significant in the multivariable analysis. Age and sex were not significantly associated with STEC (*stx* only) infection. There were no significant associations for STEC (*stx + eae*) infection (Fig 7).

**Fig 8:**
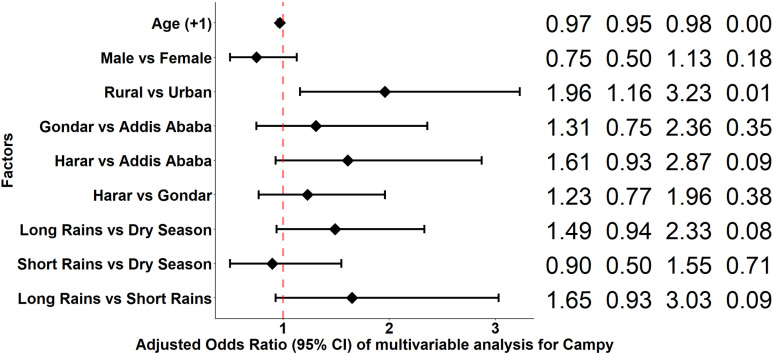
Odds Ratio of multivariable analysis of factors for *Campylobacter species.* **AOR**: Adjusted odds ratio, **LCI**: Wald lower confidence interval, **UCI**: Wald upper confidence interval.

CAMPY infection was associated with study sites, residence urbanicity, and age (Fig 8). The odds of CAMPY infection were significantly higher in Gondar and Harar compared to Addis Ababa in the univariable analysis, but this did not remain significant in the multivariable analysis. Rural residents had significantly higher odds of CAMPY infection compared to urban residents in both the univariable and multivariable analyses. The odds of CAMPY infection significantly decreased with each additional year of age in both the univariable and multivariable analyses (S2 Table). Sex and season were not significantly associated with CAMPY infection.

## Discussion

To our knowledge, this study is the first in Ethiopia to prospectively estimate the prevalence of NTS, STEC, and CAMPY in diarrheal patients concurrently across different geographic regions, seasons, and age groups. By simultaneously studying NTS, STEC, and CAMPY across diverse geographic regions and age groups, this study provides crucial insights into the interplay and burden of these pathogens in diarrheal patients and can help inform the development of more precise and effective public health interventions.

The prevalence of the targeted pathogens in our study is lower than those reported by previously conducted studies. There are several likely reasons for this. First, the majority of the previously conducted studies used biochemical and serological tests for identification of the targeted pathogens [34–39]. Such tests have lower specificity, which can lead to overestimation of the prevalence compared to the molecular characterization methods used in our study. In contrast, we used highly specific molecular diagnosis methods that provide more accurate prevalence estimates [40]. Second, our study was conducted after the emergence of COVID-19; the control measures imposed during the pandemic could have led to improved hygienic practices and changes in the consumption of raw meat, dairy products, and poultry [34,41,42]. For example, recommendations to reduce the consumption of raw meat and dairy products were part of the COVID-19 control strategy in Ethiopia, which could have resulted in a reduction of infections from the targeted pathogens [41]. Similarly, disruptions to the supply chains and markets resulted in a labor shortage that caused a decrease in poultry production, which may have contributed to reduced exposure to the targeted pathogens [42]. Third, we only tested for three pathogens. As such, the high proportion of negative results for the targeted pathogens considered in our study (i.e., 80%) does not necessarily rule out the presence of other diarrheal pathogens. For example, viruses such as noroviruses are increasingly recognized as a major cause of diarrhea, especially in foodborne outbreaks [43]. In addition, parasitic infections such as those caused by Giardia, Entamoeba histolytica, and Cryptosporidium spp. can also result in diarrhea, particularly in settings with poor sanitation and limited access to healthcare [44]. Given that our study only tested for three pathogens, it is possible that illnesses with “negative” samples were caused by viral, parasitic agents, and bacterial pathogens other than NTS, STEC, and CAMPY.

The prevalence of NTS in our study was notably lower than that reported in previous studies. A meta-analysis of 34 prospective studies conducted among diarrheal patients in Ethiopia estimated the NTS prevalence at 4.8% [35], more than three percentage points higher than our findings. However, this meta-analysis included studies that relied only on biochemical confirmation, a method that may overestimate prevalence due to its limited ability to differentiate between strains [40]. A review of 15 additional studies conducted from 2010 to 2022 among diarrheal patients in Ethiopia reported NTS prevalence ranging from 1% to 13% [36]. Two of the studies conducted in 2018 and 2020 reported a prevalence of 1%, reflecting a more recent time frame, which may better represent the current state of NTS prevalence. Our site-specific prevalence estimates were also, in general, lower than what was reported in other studies [45–49]. In Addis Ababa, our estimate for NTS (0.25%) is significantly lower than the 3.5% to 6.2% prevalence estimates reported in previous studies [39,47,50,51]. Additionally, in Northern Ethiopia (i.e., Gondar), our study found an NTS prevalence of 0.8%, which aligns with a 1.1% report by a previous study from the area [47] but is lower than that found in other studies (5.2%) [48] and (7.8%) [49]. Moreover, the 2.8% prevalence rate observed from Harar in our study is significantly lower than previous reports which ranged from 6.11% to 11.5% [45–47]. These observed differences may be attributed to variations in laboratory methods. For instance, Eguale et al. [39] and Kebede et al. [50] relied on serological tests, which can have lower specificity than the tests we used and may cross-react with non-target organisms. For example, the presence of thousands of *Salmonella* strains with closely related antigenic structures, cross-reactivity is highly possible when laboratory methods with lower specificity are used. Further, Beyene et al. [51] used biochemical identification methods, such as API 20E kits, which lack the precision, sensitivity and specificity of molecular techniques. Biochemical tests may mis-identify closely related strains of NTS due to overlapping metabolic characteristics, while serological assays, such as slide agglutination, can cross-react with non-pathogenic strains or residual antigens, leading to false positives and an overestimation of their prevalence [40]. Without PCR-based confirmation targeting specific genes, these methods may lead to an overestimation of NTS prevalence.

One of the important findings from the current study is that the prevalence of NTS varied significantly across different regions of Ethiopia. The prevalence of NTS was lower in Addis Ababa compared to Harar and Gondar, presumably due to the availability of more hygiene infrastructure and public health interventions in the capital of the country. In addition, all participants in Addis Ababa were urban residents and, therefore, may have increased exposure to public health and food safety information, and be able to implement prevention measures such as sanitation practices [37,38].

An important contribution of this study is the estimates of the prevalence of STEC, as most studies conducted in Ethiopia have only investigated specific subsets of STEC variants, such as *E. coli* O157: H7 [37–39,49]. In contrast, our study considered all STEC serotypes. Shiga toxins, encoded in genes *stx1* and *stx2* respectively, can cause bloody diarrhea and lead to life-threatening conditions like HUS and central nervous system abnormalities, especially in combination with genes coding for intimin (*eae*) [50]. While all STEC strains possess *stx* genes, only a subset of these strains also carries the *eae* gene. While over 100 serotypes of STEC (*stx* + *eae*) have been identified, the five most commonly associated with human illness are O157: H7, O26: H11, O103: H2, O111: H8, and O145: H28 [8,9]. Our analysis focused on these genes to consider the multitude of STEC serotypes, including the non-O157 strains that are often overlooked. As expected, the prevalence of STEC (*stx* only) was higher than the prevalence of STEC (*stx* + *eae*), as the latter is a subset of the former. Our study found no association between age and STEC infection, which aligns with other studies conducted among children with diarrhea in Ethiopia [20,52–54].

The prevalence of STEC, regardless of the definition used, varied across study sites. In Addis Ababa, our estimate of 3.28% for STEC (*stx* + *eae*) was higher than previous estimates of 1.6% [28] and 2.8% [55]. However, Zelelie et al. [28] only tested stool samples with blood, mucus, or pus for STEC (*stx* + *eae*) [28], whereas we considered all diarrheal stools. Notably, certain STEC serotypes can cause watery diarrhea without visible signs of blood or mucus, which could lead to underestimating the prevalence of STEC (*stx* + *eae*) [20,56]. In addition, overlapping symptoms such as blood, mucus, and pus in the stool can also be a common symptom of other bacterial pathogens such as *Shigella spp*. and *Salmonella spp*., [57] which could dominate the stool samples selected for analysis. Similarly, Gutema et al., [55] targeted only the STEC O157 serotype, which may explain our higher prevalence. In Gondar, our estimate of 7.56% for STEC (*stx* only) was similar to the estimate of 7.7% for STEC (*stx* only) from a previous study 7.7% [58]. However, our estimate of 2.87% for STEC (*stx* + *eae*) was substantially lower than a previous estimate of 11.1% for STEC (*stx* + *eae*) [59]. While both studies focused on children under the age of five, Engda et al., [59] considered children who had cattle in their households, which might have increased the likelihood of exposure and explained the higher estimates. In Harar, our estimate of 4.15% for STEC (*stx* + *eae*) was lower than the estimate of 15.3% from another prospective study among children under five years with diarrhea [20], but that study used latex agglutination tests without PCR confirmation, which has lower specificity than the test used in the current study. This may have led to the misidentification of other pathogens to be considered as STEC (*stx* + *eae*), potentially overestimating its prevalence due to cross-reactivity or detection of non-specific antigens.

The overall prevalence of CAMPY in this study was 4.46%, which is about half of previous estimates. For example, a meta-analysis of eight studies conducted between 2000 and 2010 estimated the prevalence of CAMPY to be 10% (95% CI: 7, 13) [60]. The reason for this disparity could be because the studies in the meta-analyses covered areas including Amhara, Oromia, Sidama, and Gambella regions where the majority of the participants were rural residents. In contrast, our study focused on three major cities and about 70% of the participants were urban residents Urban areas tend to have lower prevalence of CAMPY due to reduced direct exposure to animals, and increased access to treated water sources while rural residents are more likely to engage in animal-related occupations and have poorer water and sanitation infrastructure, all of which could increase exposure to CAMPY infection [61]. In fact, our study found that rural residents have significantly higher odds of CAMPY infection compared with the urban residents, which aligns with results from a previous review [62].

The prevalence of CAMPY was also associated with age, where the odds of CAMPY infection decreased by 3% for every one-year increase in age. Lengerh et al. [61] also found that children under one year had higher CAMPY prevalence compared to older children. Moreover, a meta-analysis of 15 studies conducted among children under five years in Ethiopia reported that age is significantly associated with the prevalence of CAMPY infections. Younger children, particularly those under five years old, were found to be more susceptible to these infections due to factors such as an underdeveloped immune system and higher exposure to contaminated sources [63]. Another review of 25 studies conducted among diarrheal patients found that age was associated with CAMPY prevalence where children under five years had the highest risks of CAMPY infection compared to older children with infants and toddlers under two years being particularly at increased risk, showing prevalence rates as high as 30–40% and much higher than older children [64].

Notable differences in the prevalence of CAMPY were observed between the three study sites, which may reflect differences in the status of hygiene practices and public awareness in these places [37,38]. Moreover, the exposure to reservoir animals such as chickens and cattle and consumption of unpasteurized milk might be lower in the urban areas, which might contribute to the lower prevalence in Addis Ababa [65]. However, the overall prevalence in all three sites in the current study is lower than the prevalence in the previous studies, except for one study from Addis Ababa. For example, a study of children under the age of five in Addis Ababa found the prevalence of CAMPY to be 3.2% [66], which is higher than our estimate, but used laboratory methods that likely overestimated prevalence. Another study of adults and children in Addis Ababa found the prevalence to be 1.8% [67], which is lower than our estimates. However, the estimates of both studies are within the confidence intervals of our studies. Similarly, a prospective study among children under five years with diarrheal illness in Northern Ethiopia (i.e., Gondar), estimated the prevalence of CAMPY to be 15.4% [61] while a study among diarrheal patients of all ages in Northern Ethiopia (i.e., Bahirdar), estimated the prevalence of CAMPY to be 8.0% [68]. These estimates are higher than our estimates for Gondar, but both studies used laboratory methods with low specificity. In addition, a prospective study among under five children with diarrhea in Eastern Ethiopia (i.e., Harar), estimated the prevalence of CAMPY to be 8.4% [45] which is higher than our estimates for Harar. This difference might be because approximately 50% of their study participants were from rural areas, whereas only 26% of our study participants lived in rural areas. Moreover, another prospective study among 100 children under five years with diarrhea in Harar estimated the prevalence range of 50% to 88% [18] which is significantly higher than our estimates, but they used laboratory methods with high sensitivity, but low specificity.

As expected, significant seasonal variations were observed in the prevalence rates of the three pathogens across different geographic areas. Prevalence was generally higher during the long rainy season, which could be attributed to increased runoff and subsequent contamination of water sources. Heavy rainfall often leads to the overflow of latrines and increased contamination of water bodies with pathogens from animal feces, thereby raising the risk of infection. This aligns with previous studies indicating that heavy rainfall facilitates pathogen transmission through contaminated water sources [69–72]. Interestingly, high prevalence was also observed in October, which is traditionally considered the beginning of the dry season but also characterized by transitional environmental conditions [73]. For example, the prevalence of NTS and STEC (*stx* only) peaked in October, while STEC (*stx* + *eae*) peaked in November. The high prevalence observed in October and November could be due to lags between exposure and diagnosis [74]; residual contamination of water sources; use of contaminated water sources due to reduced water availability; and increased contact with animals during the dry season [69].

Overall, this study provides valuable insights on the prevalence of the three targeted pathogens in Ethiopia. However, there are certain limitations of the current study that should be noted. First, the study population was limited to those experiencing diarrhea and submitting a stool sample to the hospital clinical laboratories. Hence, our estimates cannot be interpreted as prevalent in the general population. This is because individuals who are experiencing diarrhea and seek medical care are more likely to have an active infection and severe symptoms, which can lead to a higher detection rate of pathogens. Second, the study’s conclusions may not be generalizable to other populations and geographic areas in Ethiopia. Specifically, this study only included three cities in a country with diversified geographical and dietary practices that might contribute to foodborne illness. Moreover, different regions in Ethiopia may have varying risk factors for infection, healthcare access, and pathogen prevalence, meaning that the results from these areas may not represent the entire country. Third, during the pandemic, healthcare-seeking behavior might have changed. People may have avoided hospitals due to fear of COVID-19, which could have biased our results. Fourth, the selected hospitals could potentially introduce bias related to access and health disparities. For instance, patients from rural or underserved areas might have limited access to specialized care, which could affect the diversity of cases represented in our study. Despite these limitations, the results of this study provide important new insights into the epidemiology of these pathogens in Ethiopia.

## Conclusion

The findings from this study provide much needed evidence on the prevalence of NTS, STEC (*stx* only), STEC (*stx* + *eae*), and CAMPY across different geographic areas, seasons, and age groups in Ethiopia. Our results emphasize the need for region-specific and demographic-specific strategies to effectively reduce the burden of foodborne illnesses. Regions with higher pathogen prevalence, such as Harar, require localized strategies to enhance water quality, improve sanitation and hygiene practices, and increase public awareness of food safety. The results of this study can inform surveillance efforts and implementation of targeted interventions to reduce transmission and mitigate the impact of foodborne illnesses in Ethiopia.

## Supporting information

S1 Text Inclusivity in global research. (DOCX)

S2 Text Laboratory survey instrument. (DOCX)

S3 Text Laboratory analysis of the non-typhoidal *Salmonella* (NTS), Shiga-toxin producing *Escherichia coli* (STEC), and *Campylobacter spp*., (CAMPY). (DOCX)

S1 Table Prevalence of selected demographics and clinical information for patients with confirmed pathogens of interest. (DOCX)

S2 Table Univariable and multivariable logistic regression analysis of selected factors. (DOCX)
