# Correction: Prevalence of Shiga toxin-producing Escherichia coli, Salmonella, and Campylobacter species among diarrheal patients from three major hospitals in Ethiopia

**DOI:** 10.1371/journal.pgph.0005115

**Published:** 2025-08-22

**Authors:** Amete Mihret Teshale, Woldaregay Erku Abegaz, Binyam Moges Azmeraye, Desalegne Degefaw, Devin LaPolt, Zelalem Bonger, Alem Abrha Kalayu, Eyasu Tigabu, Lina Gazu, Getnet Yimer, Ebba Abate, Estifanos Tsige, Geremew Tasew, Yadeta Dessie, Gashaw Biks, James A. Barkley, Ariel V. Garsow, Aaron Beczkiewicz, Silvia Alonso, Barbara Kowalcyk

The 18^th^ author’s name is spelled incorrectly. The correct name is: Aaron Beczkiewicz.
