# Supplementary material for: Prevalence of Shiga toxin-producing Escherichia coli, Salmonella, and Campylobacter species among diarrheal patients from three major hospitals in Ethiopia
Source: PLOS Glob Public Health. 2025 Apr 21;5(4):e0004407. doi: 10.1371/journal.pgph.0004407 (PMC12011234; doi:10.1371/journal.pgph.0004407)
Supplement: S2 Text — (DOCX) [file pgph.0004407.s002.docx]

# S2 Text: Laboratory Survey Instrument

**A0.0** Participant ID

[ASSIGNED BY TEAM]

**A1.0** STRATUM - Region

[ASSIGNED BY TEAM]

**A2.0** STRATUM – Season

[ASSIGNED BY TEAM]

**A3.0** Date of Interview

[INSERT DATE]

**A4.0** Language of Interview

[AFAAN OROMO AMHARIC ENGLISH OTHER]

**A5.0** Name of enumerator

SELECT FROM LIST

**A6.0** Name of hospital where recruitment is done

SELECT FROM LIST

*OBTAIN CONSENT AND BEGIN INTERVIEW WITH RESPONDENT*

# Section 1: RESPONDENT PROFILE

**C1.0** What is your first name? [TEXT ENTRY]

**C1.1** Full address of the person who provides stool sample

[TEXT ENTRY]

**C1.2** Distance from the hospital

[NUMBER NETRY…ENTER 9999 IF UNKNOWN]

**C1.3 *The person who is providing the stool sample is:***

***RESPONDENT***

***OTHER individual***

***IF C1.3= “RESPONDENT” THEN SKIP TO C1.5***

**C1.4** What is the name of the individual who is providing the stool sample?

[TEXT ENTRY] →THIS RESPONSE WILL REPLACE [NAME] IN THE REST OF THE SURVEY.

If C1.3 = “RESPONDENT” then “you” replaces [NAME’s] (with appropriate corrections in Q’s)

**C1.5** What is [NAME’s] age? (YEARS)

[NUMERIC ENTRY – 9999 IF UNKNOWN/PREFER TO NOT RESPOND]

[if less then 12 months, enter 0]

IF C1.5 >15 THEN SKIP TO C1.10

IF 15> C1.5 >0 GO TO C1.6

**C1.5.1** How many months old is the child? (SELECT FROM 0 TO 11)

**C1.6** Height of [NAME] ___________ (UNITS?)

**C1.7** Weight of [NAME] ____________ (UNITS?)

**C1.8** Delivery Type for [NAME]

VAGINAL DELIVERY

C-SECTION

**C1.9** Was [NAME] breast feed?

YES

**C1.9.1** Was [NAME] exclusively breast feed?

YES

NO

**C1.9.2** When did [NAME] start weaning?

BEFORE 6 MONTHS

AFTER 6 MONTHS

NO

**C1.10** Gender? (Answer by observation)

MALE

FEMALE

**C1.11** What is [NAME’s] current marital status?

SINGLE

MARRIED

DIVORCED

WIDOWED

**C1.12** Is [NAME] the head of the household?

YES

NO

**C1.12.1** What is [NAME’s] relation to the household head

WIFE/HUSBAND

MOTHER/FATHER

BRTHER/SISTER

SON/DAUGHTER

OTHER

**C2.0** What is the highest level of education [NAME’s] have completed? (APPLIES ONLY IF CHILD>3YR)

NEVER ATTENDED

NON- FORMAL

PRE- SCHOOL

GRADES 1- 8

GRADES 9 – 10

PREPARATORY (GRADES 11-12) OR TECHNICAL SCHOOLS

DIPLOMA OR CERTIFICATE [E.G., ASSOCIATE DEGREE, VOCATIONAL] DEGREE [E.G., MD, BSC, BA, BPHARM]

POST-GRADUATE [E.G., MSC, MA, MPH, PHD]

**C2.1** WHAT IS YOUR SPECIALTY?

[TEXT ENTRY]

OTHER

*For the following questions, “HOUSEHOLD” is defined as all individuals sleeping in this house for the past 6 months, and at least 4 days per week, sharing the same resources.*

**C3.1** How many FEMALES age 15 or older are members of [NAME]’S household? [NUMERIC ENTRY – 9999 IF UNKNOWN]

**C3.2** How many FEMALES younger than 15 years old are members of [NAME]’S household? [NUMERIC ENTRY – 9999 IF UNKNOWN]

**C4.1** How many MALES age 15 or older are members of [NAME]’S household? [NUMERIC ENTRY – 9999 IF UNKNOWN]

**C4.2** How many MALES younger than 15 years old are members of [NAME]’S household? [NUMERIC ENTRY – 9999 IF UNKNOWN]

**C5.0** Which of the following describe the primary HOUSEHOLD source(s) of income (this applies to sources coming from one or more individuals belonging to the household)? [SELECT ALL THAT APPLY]

AGRICULTURE (PRIMARY PRODUCTION)

**C5.1** Which of the following best describes the HOUSEHOLD source of AGRICULTURAL income?

CROP AGRICULTURE ANIMAL AGRICULTURE MIXED AGRICULTURE

GOVERNMENT

**C5.2** Which of the following best describes the HOUSEHOLD source of GOVERNMENT income?

LABOR

ADMINISTRATIVE

CLERICAL TECHNICAL

RESEARCH

EDUCATION EXECUTIVE

LEADERSHIP

NON-GOVERNMENT (INCL. NGO)

**C5.3** Which of the following best describes the HOUSEHOLD source of NON-GOVERNMENT income?

LABOR

ADMINISTRATIVE

CLERICAL TECHNICAL

RESEARCH

EDUCATION EXECUTIVE

LEADERSHIP

PRIVATE BUSINESS – AS OWNER OR EMPLOYEE (including informal-sector businesses, small and medium enterprises,…)

**C5.4** Which of the following best describes the HOUSEHOLD source of PRIVATE BUSINESS income (e.g. selling agricultural products, running storefront)?

LABOR

ADMINISTRATIVE

CLERICAL TEHCNICAL

RESEARCH

EDUCATION EXECUTIVE

LEADERSHIP

OTHER

**C5.5** Describe OTHER sources of HOUSEHOLD income?

[TEXT ENTRY]

**C6.0** What was last month’s INCOME of the HOUSEHOLD? (all sources together)? [BIRR]

[*in the case of inconsistent monthly income, enumerator to help participants work out the most accurate monthly average*]

Less than 2000

2000 - 4000

4000 - 6000

6000 - 8000

8000 - 10000

Greater than 10000

PREFERS NOT TO SAY

UNKNOWN

**Section 2: STOOL SAMPLE**

*For the following questions, “EPISODE” is defined as a period of days where an individual experienced the current illness separated from another episode by at least 48 hours.*

**C7.0** Does [NAME] have any of the following chronic diseases? [SELECT ALL THAT APPLY]

HYPERTENSION

ASTHMA

DIABETES

CANCER

KIDNEY DISEASE

CARDIOVASCULAR DISEASE

GASTROINTESTINAL DISEASE

OTHER [TEXT ENTRY]

DON’T KNOW

**C8.0** Is the person providing the stool sample ill?

YES

NO

IF C8.0 = “NO” THEN SKIP TO **C9.0**

**C8.1** Duration of current illness: ____________________

**C8.2** What are the signs and symptoms? [SELECT ALL THAT APPLY]

LOOSE, WATERY STOOLS

ABDOMINAL CRAMPS

ABDOMINAL PAIN

FEVER

BLOOD IN THE STOOL.

MUCUS IN THE STOOL.

BLOATING

NAUSEA

OTHER [TEXT ENTRY]

**C8.3** Was the stool sample ordered due to this illness?

YES

NO

C8.3.1 Why was the stool sample ordered?

[TEXT ENTRY]

**C8.4** Is [NAME] hospitalized?

YES

NO

**C9.0** Who ordered the stool sample?

HEALTHCARE PROVIDER

**C9.1** What type of healthcare provider requested the stool sample? [SELECT ALL THAT APPLY]

GOVERNMENT HOSPITAL

GOVERNMENT CLINIC

PRIVATE HOSPITAL

PRIVATE CLINIC

HEALTH CENTER

HEALTH POST

PHARMACIST / DRUGGIST

TRADITIONAL HEALER

OTHER

SELF REQUESTED

OTHERS

**C10.0** What tests are being performed on the stool sample?

DIRECT STOOL MICROSCOPY

PARASITOLOGY

WET MOUNT

BACTERIAL CULTURE

BACTERIAL CULTURE AND ANTIBIOTIC SUSCEPTIBILITY TEST

OTHERS

**C11.0** Did [NAME] visit a healthcare provider for the current illness episode?

YES

**C11.1** What type of healthcare provider did [NAME] visit? [SELECT ALL THAT APPLY]

GOVERNMENT HOSPITAL

GOVERNMENT CLINIC

PRIVATE HOSPITAL

PRIVATE CLINIC

HEALTH CENTER

HEALTH POST

PHARMACIST / DRUGGIST

TRADITIONAL HEALER

OTHER [TEXT ENTRY]

**C11.2** How far is the facility from the household dwelling?

[NUMERIC ENTRY – 9999 IF UNKNOWN]

**C11.3** Indicate the unit of the reported distance

KM

METRES

HOURS

MINUTES

PRICE PAID

OTHER

**C11.3.1** What UNIT is the reported distance?

[TEXT ENTRY]

NO

**C11.4** Which of the following factors (if any) prevented [NAME] from visiting a healthcare provider for the current illness episode? [SELECT ALL THAT APPLY]

PERSONAL CHOICE

COST OF VISIT

NO ACCESS TO HEALTHCARE PROVIDER OTHER

UNKNOWN

OTHER

**C11.4.1** What other factors prevented [NAME] from visiting a healthcare provider for the current illness episode?

[TEXT ENTRY]

DON’T KNOW

**C12.0** Did [NAME] receive or take any treatment for the current illness episode?

YES

**C12.1** Which of the following were used to treat [NAME]’s current illness episode? [SELECT ALL THAT APPLY] (once a participant is allowed to provide his/her answer, interviewer to read aloud options that the person may have missed).

ORAL FLUIDS TO PREVENT DEHYDRATION

INTRAVENOUS FLUIDS FOR REHYDRATION

MEDICINE TO RELIEVE PAIN

MEDICINE TO STOP DIARRHEA

MEDICINE TO STOP FEVER

MEDICINE UNKNOWN PURPOSE

ANTIBIOTICS

ANTIPARASITIC/ANTIPROTOZOA

NATURAL/TRADITIONAL REMEDIES (RICE, FETO, GARLIC, GINGER, MINERAL WATER, etc..)

DON’T KNOW

OTHER

**C12.2** How was the medicine obtained? [SELECT ALL THAT APPLY]

HEALTHCARE PROVIDER PRESCRIBED

**C12.2.1** Did [NAME] take this medicine for the full duration recommended?

YES

NO

NOT APPLICABLE (THERE IS NO RECOMMENDED DURATION)

DON’T KNOW

OTHER

PRESCRIBED BY A TRADITIONAL HEALER

SELF-PRESCRIBED – OBTAINED FROM LOCAL DISPENSARY

SELF-PRESCRIBED – MEDICINE REMAINING FROM PREVIOUS ILLNESS SELF-PRESCRIBED – OBTAINED FROM ANOTHER INDIVIDUAL

SELF-PRESCRIBED – MADE AT HOME

DON’T KNOW

OTHER

NO

**C12.3** Which of the following factors (if any) prevented [NAME] from treating the current illness episode? [SELECT ALL THAT APPLY]

PERSONAL CHOICE

COST OF MEDICINE

MEDICINE UNAVAILABLE AT PHARMACY/ DISPENSARY

PHARMACY UNAVAILABLE IN THE AREA

OTHER

**C12.3.1** What other factors prevented [NAME] from treating the current illness episode?

[TEXT ENTRY]

DON’T KNOW

**C13.0** How many separate/unrelated episodes of DIARRHEA has [NAME] experienced in the past four (4) weeks?

[NUMERIC ENTRY – 9999 IF UNKNOWN]

**C14.0** How many separate/unrelated episodes of BLOODY STOOLS has [NAME] experienced in the past four (4) weeks presented alongside diarrhea?

[NUMERIC ENTRY – 9999 IF UNKNOWN]

**C15.0** How many separate/unrelated episodes of VOMITING (of suspected infectious disease origin) has [NAME] experienced in the past four (4) weeks?

[NUMERIC ENTRY – 9999 IF UNKNOWN]

**C16.0** How many separate/unrelated episodes of FEVER (of suspected infectious disease origin) has [NAME] experienced in the past four (4) weeks?

[NUMERIC ENTRY – 9999 IF UNKNOWN]

**C17.0** How many separate/unrelated episodes of ABDOMINAL PAIN (of suspected infectious disease origin) has [NAME] experienced in the past four (4) weeks?

[NUMERIC ENTRY – 9999 IF UNKNOWN]

**Section 3: COSTS**

*For the following questions, “EPISODE” is defined as a period of days where an individual experienced the current illness separated from another episode by at least 48 hours.*

**C18.0** What was the total out-of-pocket cost of HEALTHCARE PROVIDER VISITS for [NAME]’s current illness episode? (excluding cost of medicines)

[NUMERIC ENTRY – BIRR, 0 if PROVIDED FOR FREE]

**C19.0** What was the total out-of-pocket cost of TRAVEL, TRANSPORTATION AND LODGING TO HEALTHCARE PROVIDER for [NAME]’s current illness episode?

[NUMERIC ENTRY – BIRR, 0 if PROVIDED FOR FREE]

**C20.0 ONLY APPLIES IF C17.0=”YES”**

**C20.0** What was the total out-of-pocket cost of MEDICINE AND OTHER TREATMENT for [NAME]’s current illness episode?

[NUMERIC ENTRY – BIRR, 0 if PROVIDED FOR FREE]

**C21.0** Were there ANY (OTHER) OUT-OF-POCKET COSTS for [NAME]’s current illness episode?

YES

**C21.1** What were other out-of-pocket costs for [NAME]’s current illness episode?

[TEXT ENTRY]

**C21.2** What was the total cost of other out-of-pocket expenses for [NAME]’s current illness?

[NUMERIC ENTRY – BIRR, 0 if PROVIDED FOR FREE]

NO

DON’T KNOW

**C22.0** Does [NAME] attend school?

YES

**C22.1** Did [NAME] miss any days of school due to [NAME]’s current illness episode?

YES

**C22.1.1** How many days of school were missed by [NAME] as a result of [NAME]’s current illness episode?

[NUMBERIC ENTRY-0 IF NONE, 9999 IF UNKNOWN]

NO

NO

**C23.0 *Does [NAME] work?***

YES

**C23.1** Did [NAME] miss any days of work due to [NAME]’s current illness episode?

YES

**C23.1.1** How many days of WORK were missed by [NAME] because of [NAME]’s current illness episode?

[NUMERIC ENTRY –0 IF NONE, 9999 IF UNKNOWN]

NO

**C23.2** How many days per month does [NAME] typically work?

[NUMERIC ENTRY – 99 IF UNKNOWN]

**C23.3** What is [NAME]’s main occupation?

[TEXT ENTRY]

**C23.4** Where did [NAME] do their work?

Business House

Office

At home

On street

‘Gulit’/open market

Farm/field

Factory

Quarry or mine

Anywhere as found

Where customer available

Construction site

Lakes/rivers/wells

Other: [TEXT ENTRY]

**C23.5** What was the major product or service of this organization?

[TEXT ENTRY]

**C23.6** What was [NAME]’s employment status?

Employee: Government or NGO

Employee: Private Org.

Employer

Self-Employed

Unpaid Family Worker

Other: [TEXT ENTRY]

**C23.7** What is [NAME]’s typical MONTHLY INCOME?

LESS THAN 2000

2000 - 4000

4000 - 6000

6000 - 8000

8000 - 10000

GREATER THAN 10000

UNKNOWN

PREFER NOT TO SAY

NO (GO TO QUESTION C48.0)

DON’T KNOW

**C24.0** Has [NAME] been assisted by a caregiver during the current illness episode?

YES

**C24.1** Does [NAME]’S caregiver have a paid job?

YES

**C24.1.1** Did [NAME]’s caregiver miss any days of work due to [NAME]’s current illness episode?

YES

**C24.1.1.2** How many days of WORK were missed by [NAME]’s caregiver because of [NAME]’s current illness episode?

[NUMERIC ENTRY –0 IF NONE, 9999 IF UNKNOWN]

**C24.1.2** What is [NAME]’s caregiver’s main occupation?

[TEXT ENTRY]

**C24.1.3** Where does [NAME] ’s caregiver work?

Business House

Office

At home

On street

‘Gulit’/open market

Farm/field

Factory

Quarry or mine

Anywhere as found

Where customer available

Construction site

Lakes/rivers/wells

Other: [TEXT ENTRY]

**C24.1.4** What was the major product or service of this organization?

[TEXT ENTRY]

**C24.1.5** What was [NAME]’s caregiver’s employment status?

Employee: Government or NGO

Employee: Private Org.

Employer

Self-Employed

Unpaid Family Worker

Other: [TEXT ENTRY]

**C24.1.6** What is [NAME]’s caregiver’s typical MONTHLY INCOME (all sources together)? [BIRR]

[in the case of inconsistent monthly income, enumerator to help participants work out the most accurate monthly average]

Less than 2000

2000 - 4000

4000 - 6000

6000 - 8000

8000 - 10000

Greater than 10000

PREFERS NOT TO SAY

NO INCOME

UNKNOWN

**C24.1.7** What is [NAME]’s caregiver typical hourly rate?

[NUMERICAL ENTRY] 999 IF UNKNOWN; 998 IF PREFER NOT TO SAY

**C24.1.8** How many days per month does [NAME]’s caregiver typically work?

[NUMERICAL ENTRY – 99 IF UNKNOWN]

NO

DON’T KNOW

**C24.2** Does [NAME]’S caregiver attend school?

YES

**C24.2.1** How many days of SCHOOL were missed by [NAME]’s caregiver because of [NAME]’s current illness episode?

[NUMERIC ENTRY –0 IF NONE, 9999 IF UNKNOWN]

NO

DON’T KNOW

NO

DON’T KNOW

# Section 4: ENVIRONMENTAL EXPOSURES

*The following questions pertain to the entire household as previously defined.*

[REPEAT QUESTIONS CX.0-CX.3 FOR EACH ANIMAL]

**C25** CATTLE

**C26** GOATS

**C27** SHEEP

**C28** CHICKENS, DUCKS, or OTHER POULTRY

**C29** DOGS or CATS

[LOOP - START]

**CX.0** Do you have [INSERT ANIMAL] in your household?

YES

**CX.1** Where are the [INSERT ANIMAL] kept?

INSIDE HOUSE OUTSIDE HOUSE

DON’T KNOW

**CX.2** For what purpose are the [INSERT ANIMAL] kept?

INCOME TRADE CONSUMPTION SAVINGS TRACTION

PET

DON’T KNOW

OTHER [TEXT ENTRY]

**CX.3** Who cares for the [INSERT ANIMAL] of the household? [SELECT ALL THAT APPLY]

FEMALE HEAD OF FAMILY

OTHER FEMALE ADULTS IN HOUSEHOLD

OTHER FEMALE CHILDREN IN HOUSEHOLD

MALE HEAD OF FAMILY

OTHER MALE ADULTS IN HOUSEHOLD

OTHER MALE CHILDREN IN HOUSEHOLD

NOT CARED for by household members

DON’T KNOW

NO

**CX.4** Have any household members been in contact with [INSERT ANIMAL] in the past four (4) weeks OUTSIDE OF THE HOUSEHOLD (i.e., due to their occupation, or else)?

YES

**CX.5** What household member has been in contact with [INSERT ANIMAL]? [SELECT ALL THAT APPLY]

FEMALE HEAD OF FAMILY

OTHER FEMALE ADULTS IN HOUSEHOLD OTHER FEMALE CHILDREN IN HOUSEHOLD MALE HEAD OF FAMILY

OTHER MALE ADULTS IN HOUSEHOLD OTHER MALE CHILDREN IN HOUSEHOLD

DON’T KNOW

NO

[LOOP - FINISH]

**C30.0** What is the primary source of water for the household? SELECT ALL THAT APPLY (BUT IT MUST BE PRIMARY SOURCES)

PIPED INTO DWELLING/HOUSE

PIPED INTO YARD COMMUNAL TAP NEIGHBOR’S HOUSE PROTECTED WELL UNPROTECTED WELL PROTECTED SPRING UNPROTECTED SPRING

SURFACE WATER [e.g., RIVER, LAKE, CANAL] RAINWATER

TANKER TRUCK

BOTTLED WATER

FILTERED WATER

OTHER [TEXT ENTRY]

DON’T KNOW

**C31.0** What type of toilet is present in the household?

FLUSH TO PIPED SEWER SYSTEM

FLUSH TO SEPTIC TANK

PIT LATRINE WITH COVER

PIT LATRINE WITHOUT COVER

NO LATRINE FACILITY / OPEN FIELD

OTHER [TEXT ENTRY]

DON’T KNOW

**SECTION 5: FOOD CONSUMPTION AND PREPARATION PRACTICES**

**C32.0** Where do you get your GRAINS AND DRY GOODS for the household? [SELECT ALL THAT APPLY]

SELF-GROWN

FARMER OTHER THAN SELF

LOCAL CENTRAL MARKET

SMALL SHOP

SELF-SERVICE GROCERY

SUPERMARKET

DON’T KNOW

OTHER [TEXT ENTRY]

**C33.0** Where do you get your ANIMAL SOURCE PRODUCTS such as eggs, meat, milk, and cheese for the household? [SELECT ALL THAT APPLY]

SELF-GROWN

FARMER OTHER THAN SELF

LOCAL CENTRAL MARKET

SMALL SHOP

SELF-SERVICE GROCERY

SUPERMARKET

DON’T KNOW

OTHER [TEXT ENTRY]

**C34.0** Where do you get your FRUIT AND VEGETABLES for the household? [SELECT ALL THAT APPLY]

SELF-GROWN

FARMER OTHER THAN SELF

LOCAL CENTRAL/STREET MARKET (INCL. ‘GULIT’)

SMALL SHOP

SELF-SERVICE GROCERY SUPERMARKET

DON’T KNOW

OTHER [TEXT ENTRY]

**C35.0** Is there a dedicated area in the residence where ALL FOOD is prepared?

YES

**C35.1** Are animals prevented from entering this area?

YES

NO

NO

**C36.0** Who USUALLY (I.E. MOST COMMONLY) prepares food for the household? [SELECT ALL THAT APPLY]

RESPONDENT

INDIVIDUAL EMPLOYED BY HOUSEHOLD

FEMALE HEAD OF FAMILY

OTHER FEMALE ADULTS IN HOUSEHOLD

OTHER FEMALE CHILDREN IN HOUSEHOLD

MALE HEAD OF FAMILY

OTHER MALE ADULTS IN HOUSEHOLD

OTHER MALE CHILDREN IN HOUSEHOLD

**C37.0** Describe briefly how you regularly prepare a dish containing VEGETABLES OR FRUITS. Please ensure you tell me all steps you would do from the moment you start cooking. (Select All that are mentioned)

WASH HANDS PRIOR TO HANDLING FOOD

WASH HANDS AFTER TOUCHING RAW FOOD

WASH SURFACES PRIOR TO HANDLING FOOD

WASH UTENSILS BEFORE FOOD PREPARATION

BOIL OR FILTER WATER USED TO PREPARE FOOD

WASH FRUITS AND VEGETABLES

DISINFECT FRUITS AND VEGETABLES (I.E. INCLUDING SOAKING WITH SALT, OR A FOOD-GRADE DISINFECTANT)

PREPARE UNCOOKED MEAT AND VEGETABLES WITH SEPARATE UTENSILS MEASURE TEMPERATURE OF COOKED FOODS

NONE OF THE ABOVE APPLY

DON’T KNOW

OTHER

**C37.0** Describe briefly how you regularly prepare a dish containing ANIMAL SOURCE FOODS. Please ensure you tell me all steps you would do from the moment you start cooking.(Select All that are mentioned)

WASH HANDS PRIOR TO HANDLING FOOD

WASH SURFACES PRIOR TO HANDLING FOOD

BOIL OR FILTER WATER USED TO PREPARE FOOD

WASH FRUITS AND VEGETABLES

DISINFECT FRUITS AND VEGETABLES (I.E. INCLUDING SOAKING WITH SALT, OR A FOOD-GRADE DISINFECTANT)

PREPARE UNCOOKED MEAT AND VEGETABLES WITH SEPARATE UTENSILS

MEASURE TEMPERATURE OF COOKED FOODS

NONE OF THE ABOVE APPLY

DON’T KNOW

OTHER

**C38.0** Which of the following food handling practices are used IN ADDITION TO THE ONES MENTIONED ABOVE within the household when preparing food? [SELECT ALL THAT APPLY] Prompt only the practices from this list that were not mentioned in the previous 2 questions.

WASH HANDS PRIOR TO HANDLING FOOD

WASH HANDS AFTER TOUCHING RAW FOOD

WASH SURFACES PRIOR TO HANDLING FOOD

BOIL OR FILTER WATER USED TO PREPARE FOOD

WASH FRUITS AND VEGETABLES BEFORE PREPARATION

PREPARE UNCOOKED MEAT AND VEGETABLES WITH SEPARATE UTENSILS

MEASURE TEMPERATURE OF COOKED FOODS

NONE OF THE ABOVE APPLY

UNKNOWN

**C39.0** Do you treat the water you use to wash your hands when cooking?

YES

**C39.1** How do you treat the water?

Filtering

Boiling

Chemicals (disinfectant, tablets,…)

(…OTHER TO BE FILLED IN BY ENUMERATORS)

NO

DON’T KNOW

**C40.0** Does the household have a refrigerator?

YES

**C40.1** Does the household have consistent electricity?

CONSISTENT ELECTRICITY INCONSISTENT ELECTRICITY

**C40.2** If the time between purchase of meat and preparation is greater than 1 hour, do you store the meat in your refrigerator?

YES NO

NO

[REPEAT QUESTIONS CX.0-CX.3 FOR EACH FOOD]; IF CX.0 = “NO” THEN SKIP TO NEXT FOOD

|  | Past 7 DAYS | | LAST TIME CONSUMED | |
| --- | --- | --- | --- | --- |
|  | CX.0 Was [INSERT FOOD] consumed by [NAME of person last sick] in the past 7 days (in or outside the house)? | CX.1 How often did [NAME of person last sick] consumed [INSERT FOOD] in the past 7 days? | CX. 2 Where did [NAME of person last sick] consumed this last time? (only for beef and dairy) | CX. 3 How was it prepared? Focus on the last time consumed? |
| **C41.** CATTLE MILK | Yes /No | Every meal in a day  At least one meal everyday  More than 4 days  Less than 4 days | At home  At a market, restaurant  Location other than home | Raw (meaning not boiled/cooked, not pasteurized)  Boiled/cooked (but not pasteurized)  Pasteurized (but not boiled/cooked)  Pasteurized and boiled/cooked |
| **C42.** MILK FROM CAMELS | Yes /No | Every meal in a day  Everyday  More than 4 days  Less than 4 days | At home  At a market, restaurant  Location other than home | Raw (meaning not boiled/cooked, not pasteurized)  Boiled/cooked (but not pasteurized)  Pasteurized (but not boiled/cooked)  Pasteurized and boiled/cooked |
| **C43.** MILK FROM SHEEP AND GOATS | Yes /No | Every meal in a day  Everyday  More than 4 days  Less than 4 days | At home  At a market, restaurant  Location other than home | Raw (meaning not boiled/cooked, not pasteurized)  Boiled/cooked (but not pasteurized)  Pasteurized (but not boiled/cooked)  Pasteurized and boiled/cooked |
| **C44.** YOGURT | Yes /No | Every meal in a day  Everyday  More than 4 days  Less than 4 days | At home  At a market, restaurant  Location other than home | Raw (meaning not boiled/cooked, not pasteurized)  Boiled/cooked (but not pasteurized)  Pasteurized (but not boiled/cooked)  Pasteurized and boiled/cooked |
| **C45.** FERMENTED MILK | Yes /No | Every meal in a day  Everyday  More than 4 days  Less than 4 days | At home  At a market, restaurant  Location other than home | Raw (meaning not boiled/cooked, not pasteurized)  Boiled/cooked (but not pasteurized)  Pasteurized (but not boiled/cooked)  Pasteurized and boiled/cooked |
| **C46.** ANY OTHER FOOD MADE FROM MILK | Yes /No | Every meal in a day  Everyday  More than 4 days  Less than 4 days | At home  At a market, restaurant  Location other than home | Raw (meaning not boiled/cooked, not pasteurized)  Boiled/cooked (but not pasteurized)  Pasteurized (but not boiled/cooked)  Pasteurized and boiled/cooked |
| **C47.** BEEF | Yes /No | Every meal in a day  Everyday  More than 4 days  Less than 4 days | At home  At a market, restaurant  Location other than home | Uncooked  Cooked |
| **C48.** LAMB | Yes /No | Every meal in a day  Everyday  More than 4 days  Less than 4 days |  |  |
| **C49.** GOAT | Yes /No | Every meal in a day  Everyday  More than 4 days  Less than 4 days |  |  |
| **C50.** CHICKEN | Yes /No | Every meal in a day  Everyday  More than 4 days  Less than 4 days |  |  |
| **C51.** EGGS | Yes /No | Every meal in a day  Everyday  More than 4 days  Less than 4 days |  |  |
| **C52.** LIVER, KIDNEY, HEART OR OTHER ORGAN MEATS | Yes /No | Every meal in a day  Everyday  More than 4 days  Less than 4 days |  |  |
| **C53.** INJERA, OR OTHER STAPLE FOODS MADE FROM GRAINS SUCH AS TEFF OR MAIZE | Yes /No | Every meal in a day  Everyday  More than 4 days  Less than 4 days |  |  |
| **C54.** FRUITS (MANGOES, PAPAYAS, AVOCADOS, BANANAS ORANGES, LEMONS, LIMES, OR OTHER CITRUS, ,) | Yes /No | Every meal in a day  Everyday  More than 4 days  Less than 4 days |  |  |
| **C55.** FRESH HERBS (I.e., consumed not just as spices) | Yes /No | Every meal in a day  Everyday  More than 4 days  Less than 4 days |  |  |
| **C56.** LETTUCE OR OTHER LEAFY GREENS EATEN RAW | Yes /No | Every meal in a day  Everyday  More than 4 days  Less than 4 days |  |  |
| **C57.** SPINACH OR OTHER LEAFY GREENS EATEN COOKED | Yes /No | Every meal in a day  Everyday  More than 4 days  Less than 4 days |  |  |
| **C58.** CABBAGE | Yes /No | Every meal in a day  Everyday  More than 4 days  Less than 4 days |  |  |
| **C59.** TOMATOES | Yes /No | Every meal in a day  Everyday  More than 4 days  Less than 4 days |  |  |
| **C60.** ONIONS | Yes /No | Every meal in a day  Everyday  More than 4 days  Less than 4 days |  |  |
| **C61.** CARROTS, YAMS, OR ANY OTHER FOODS MADE FROM ROOTS OR TUBERS | Yes /No | Every meal in a day  Everyday  More than 4 days  Less than 4 days |  |  |
| **C62.** ANY FOODS MADE FROM BEANS, PEAS, OR LENTILS | Yes /No | Every meal in a day  Everyday  More than 4 days  Less than 4 days |  |  |
| **C63.** NUTS | Yes /No | Every meal in a day  Everyday  More than 4 days  Less than 4 days |  |  |
| [LOOP – END] |  |  |  |  |

# Section 6: KNOWLEDGE ABOUT FOODBORNE DISEASE and FOOD SAFETY

**C64.0** Which of the following statements do you agree with?

Food and water are needed for nourishment and **cannot** make people sick.

Food and water are needed for nourishment, **but** they **can** **also** make people sick.

*The next questions will talk about the* ***safety of food****, which means making food safe to eat so that it* ***will not make us sick*** *when we eat it.*

**C65.0** Which of the following statements do you agree with?

WASHING HANDS BEFORE PREPARING FOOD IS A GOOD PRACTICE TO KEEP FOOD SAFE.

HANDWASHING BEFORE PREPARING FOOD HAS NOTHING TO DO WITH KEEPING FOOD SAFE.

**C66.0** Which of the following statements do you agree with? (Refers to vegetables that are normally consumed cooked, not to those that are normally eaten uncooked.)

EATING UNCOOKED VEGETABLES IS **SAFER** THAN EATING COOKED VEGETABLES.

EATING UNCOOKED VEGETABLES IS **AS SAFE** AS EATING COOKED FOOD.

EATING UNCOOKED VEGETABLES IS **LESS SAFE** THAN EATING COOKED VEGETABLES.

**C67.0** Which of the following statements do you agree with?

BOILING OR COOKING FOOD NORMALLY MAKES IT **LESS SAFE** TO EAT.

BOILING OR COOKING FOOD NORMALLY HAS **NO IMPACT** ON SAFETY.

BOILING OR COOKING FOOD NORMALLY MAKES IT **MORE SAFE** TO EAT.

**C68.0** What resources do you use to learn more about foodborne disease symptoms and treatment? [SELECT ALL THAT APPLY]

HEALTHCARE PROVIDERS

GOVERNMENT AGENCY [INCLUDING WEBSITES]

INTERGOVERNMENTAL AGENCY [e.g., WHO (INCLUDING WEBSITES)]

NONPROFIT OR RELIEF AGENCY [INCLUDING WEBSITES]

INTERNET SITES [NONGOVERNMENTAL]

INFORMAL SOURCES (NEIGHBORS, FAMILIES, FRIENDS….)

TV AND RADIO

SOCIAL MEDIA

POSTERS, FLYERS

OTHER

DON’T KNOW
