# Supplementary material for: Prevalence of Shiga toxin-producing Escherichia coli, Salmonella, and Campylobacter species among diarrheal patients from three major hospitals in Ethiopia
Source: PLOS Glob Public Health. 2025 Apr 21;5(4):e0004407. doi: 10.1371/journal.pgph.0004407 (PMC12011234; doi:10.1371/journal.pgph.0004407)
Supplement: S3 Text — (DOCX) [file pgph.0004407.s003.docx]

**S3 Text: Laboratory Analysis of the Non-typhoidal Salmonella (NTS), Shiga-toxin producing *Escherichia coli* (STEC), and *Campylobacter spp*., (CAMPY).**

1. **Isolation and Identification**
   1. **Identification of *Salmonella species***

Stool samples were inoculated using the Selenite-F-broth enrichment broth and incubated aerobically at 35+2^o^C overnight. Samples of the enriched broth were sub-cultured on the MacConkey (MAC, Difco^TM^, USA) and Xylose-Lysine-Deoxycholate (XLD, Difco^TM^, USA) agar plates and incubated aerobically at 35+2^o^C overnight. The plates were examined for the presence of hydrogen-sulfide producing non-fermenting colonies (reddish colony with and without black center) on XLD, and moist colorless colonies on MAC agar. Identification of SALM were conducted using biochemical tests i.e., Triple Sugar Iron Agar (TSIA, Difco™, USA), Lysine Iron Agar (LIA, Difco™, USA), Simmon Citrate Agar (SCA, Difco™, USA), Urease Agar (UA, Difco™, USA), and Sulfide Indole Motility Agar (SIMA, Difco™, USA). The genus Salmonella were confirmed using Magnetic Induction Cycler qPCR (MIC qPCR) by targeting the *invA* gene.

- 1. **Identification of Shiga-toxin Producing *Escherichia coli***

Stool samples were plated on to chromogenic media (ChromSTEC agar, France), and incubated aerobically at 35+2^o^C overnight. The presumptive STEC positive results were detected, if any mauve colonies (as described by media manufacturer) were present on the ChromSTEC agar. Confirmation of STEC was conducted using MIC qPCR machine by targeting (s*tx1*/*stx2*) and *eae* genes according to the ISO/TS 13136 guideline (ISO/TS 13136.,2012) and manufacturer’s instruction*.*

- 1. **Detection of Campylobacter species**

Detection of CAMPY were conducted using an immunoassay technique (Campy Quick Check, USA) based on the manufacturer’s instruction ([TECHLAB.,2020](https://www.techlab.com/diagnostics/foodborne-pathogens/campylobacter-diagnostics/campylobacter-quik-chek/)). The kit was examined after 10minute for the formation of purple lines in the control and test area of the kit, and report as presumptive CAMPY positive according to the manufacturer’s instruction. The immunoassay method was used for the detection of the *C. coli*, *C. jejuni*, *C. lari*, and *C. upsaliensis* species. CAMPY positive stool samples were stored at –80^o^C and used for the molecular confirmation by using the MIC qPCR machine and targeting the Campylobacter species specific 16S rRNA genes.

1. **Molecular Confirmation**
   1. **DNA Extraction: Extraction from Pure Bacterial Colony**

Extraction of DNA for the NTS and STEC confirmation were conducted from the pure colony using the boiling methods. Frozen presumed NTS and STEC isolates were sub-cultured on to the Trypticase-soya agar (TSA), incubated aerobically at 35+2^o^C overnight. Then, approximately 5 pure colonies from the TSA were suspended into 250µl of sterile nuclease free water in the Eppendorf tube to have suspension. The colony suspension was centrifuged at 14000 rpm for 10 minutes. The supernatants were discarded, and 250µl of nuclease free water were added to the cell pellets, followed by boiling in preheated heat block at 100^o^C for 10 minutes. Then, the suspension was cooled down for 15 minutes, and centrifuged at 14000 rpm for 10 minutes, and the supernatants were transferred to new sterile Eppendorf tube, dilute 1:5, and used as DNA template for our PCR testing. The amount and purity of the extracted DNA were measured with a NanoDrop apparatus (Thermo Fisher Scientific, Waltham. Massachusetts, United States).

- 1. **DNA Extraction: Extraction from The Stool Samples**

For the CAMPY confirmation, the DNA extraction was done from the frozen stool samples using the Fast Stool DNA Kit (Qiagen GmbH, Hilden, Germany) according to the manufacturer’s protocol (QIAamp® Fast DNA Stool Mini Handbook, Feb,2020). Frozen stool samples, weighing approximately 180–220 mg was placed in a 2 ml microcentrifuge tube and placed on ice. Then, 1 ml InhibitEX Buffer was added into each stool sample, and vortexed continuously for 1 min or until the stool sample was thoroughly homogenized, followed by heating the suspension for 5 min at 70-95°C, and vortexed for 15 s. The suspension was centrifuged at 12000 rpm for 1 min to pellet stool particles, and 200 µl supernatant were transferred to the tube containing 15 µl proteinase K, and 200 µl Buffer AL were added and vortexed for 15 s. The mixture was incubated at 70°C for 10 minutes, followed by addition of 200 µl of ethanol (96–100%) to the lysate, and mixed by vortexing, transfer all the lysates prepared so far (600µl lysate) into the QIAamp spin column, close the cap and centrifuge at full speed for 1 minute, repeated this step 2x to remove debris from the DNA suspension. Then, 500 µl of the wash buffer AW1 and AW2 were added stepwise, and pipet 200 µl Buffer ATE directly onto the QIAamp membrane, incubated for 1 minute at room temperature, then centrifuged at full speed for 1 minute to elute DNA, and this suspension was diluted 1:5, and used as DNA template for the PCR analysis. The amount and purity of the extracted DNA were measured with a NanoDrop apparatus (Thermo Fisher Scientific, Waltham. Massachusetts, United States).

- 1. **PCR testing (MIC qPCR Analysis)**

The PCR confirmation of the NTS, STEC, and the CAMPY were conducted using the MIC qPCR machine ([MIC qPCR Guideline](https://biomolecularsystems.com/mic-pcr/#:~:text=The%20Mic%20PCR%20machine%20uses,fan%20forced%20air%20for%20cooling.)). Besides, the ISO 6579:2002, and ISO/TS 13136.,2012 guidelines were also used for NTS and STEC confirmation respectively.

**Table 1. Primers and Probes used for PCR confirmation of Non-typhoidal *Salmonella*, Shiga-toxin Producing *Escherichia coli*, and *Campylobacter spp*.**

| **Bacteria** | **Target gene** | **Amplicon Size (bp)** | **Final Concentration (nM)** | **Primer and Probe Sequence** | **References** |
| --- | --- | --- | --- | --- | --- |
| Non-typhoidal *Salmonella* | *invA* |  | 300 | Forward (Sal1598): AACGTGTTTCCGTGCGTAAT | Heymans et al.,2018 |
|  |  |  | 300 | Reverse (Sal1859): TCCATCAAATTAGCGGAGGC |  |
|  |  |  | 150 | Probe: [6FAM] TGGAAGCGCTCGCATTGTGG[BHQ1] |  |
| Shiga-toxin Producing *Escherichia coli* | *stx1* | 131 | 300 | Forward: TTTGTYACTGTSACAGCWGAAGCYTTACG | Perelle et al.,2004 |
|  |  |  | 300 | Reverse: CCCCAGTTCARWGTRAGRTCMACRTC |  |
|  |  |  | 150 | Probe: [6FAM] CTGGATGATCTCAGTGGGCGTTCTTATGTAA[BHQ1] |  |
|  | *stx2* | 128 | 300 | Forward: TTTGTYACTGTSACAGCWGAAGCYTTACG |  |
|  |  |  | 300 | Reverse: CCCCAGTTCARWGTRAGRTCMACRTC |  |
|  |  |  | 150 | Probe: [HEX]TCGTCAGGCACTGTCTGAAACTGCTCC[BHQ1] |  |
|  | *eae* | 102 | 300 | Forward: CATTGATCAGGATTTTTCTGGTGATA |  |
|  |  |  | 300 | Reverse: CTCATGCGGAAATAGCCGTTA |  |
|  |  |  | 150 | Probe: [Cyanine5] ATAGTCTCGCCAGTATTCGCCACCAATACC[BHQ2] |  |
| *Campylobacter spp*., | *16S rRNA* |  | 300 | Forward: GATGACACTTTTCGGAGCGTAA | Platts-Mills et al, 2014 |
|  |  |  | 300 | Reverse: GCTTGCACCCTCCGTATTACC |  |
|  |  |  | 150 | Probe: [6FAM] CGTGCCAGCAGCC[BHQ1] |  |
| IAC |  | 100 | 300 | Forward: AGTTGCAGTGTAACCGTCATGT |  |
|  |  |  | 300 | Reverse: TCGACGAGACTCTGCTGTTAAG |  |
|  |  |  | 150 | Probe: AGTTGCAGTGTAACCGTCATGTACCAGTAATCTGCGTCGCACGTGTGC  ACCTAGTCTAATCACTTATGACTCAGATAACTTAACAGCAGAGTCTCGTCGA |  |

**Note:** “W” and “Y” in our sequence are ambiguous nucleotides. “W” stands for “A” or “T”, and “Y” stands for “G” or “C”. In the sequence of (stx) In the sequence Y is (C, T), S is (C, G), W is (A, T), R is (A, G), M is (A, C)

- - 1. **Non-typhoidal Salmonella Confirmation**

NTS was confirmed using monoplex PCR test by targeting the Salmonella-specific *invA* gene, and the specific primers pairs and probe sequences (Table1). A total volume of 20µl was used through a combination of the primer pair (300nm,1µl each) for the *invA* gene and internal amplification control (IAC), dual fluorescent labeled probes (150nm, 1µl), IAC template (0.075 pg/m/0.002 pg, 1µl), and master mix (1X, 10µl), nuclease free water (2µl), and finally the DNA template (1µl each) was added. The assay includes a one-cycle initial denaturation stage of 95^o^C for 2 minutes, followed by the denaturation of 95^o^C for 10 seconds, and annealing/extension steps at the 60^o^C for 60 seconds for a total of 50 cycles. The IAC was included in the PCR assays to detect the presence of PCR inhibitors and reduce false-negative results, positive control was conducted using the *S. enteritidis* (ATCC® 14028) serotype and nuclease-free water was used as a negative control.

- - 1. **Shiga toxin-producing *Escherichia coli* Confirmation**

The multiplex qPCR was used for the detection of *stx1*, *stx2*, and *eae* genes of STEC for confirmation using the assay protocol, sequences of primer, and probes (Table 1). A total volume of 20µl was used through a combination of the primer pair (300nm,1µl each), dual fluorescent labeled probes (150nm, 1µl), and master mix (1X, 10µl), and finally the DNA template(1µl) were added. The assay includes a one-cycle of initial denaturation stage of 95^o^C for 2 minutes, followed by the denaturation stage of 95^o^C for 10 seconds, and annealing/extension stage of 60^o^C for 10 seconds for a total of 50 cycles. The positive and negative control testing were conducted using the *E. coli* O157:H7 (ATCC 35150) and nuclease-free water respectively.

- - 1. **Campylobacter Species Confirmation**

The confirmation of CAMPY was conducted using the multiplex MIC qPCR using an assay protocol using the sequence of primers and probes (Table 1). The assay condition of one-cycle pre-amplification stage (initial denaturation) at 95^o^C for 10 minutes, followed by the denaturation of 95^o^C for 15 seconds and annealing/extension 55^o^C for 60 seconds with a total of 50 cycles. a total of 20µl reaction volume was used with the composition of primer pairs (1µl each), probe (1µl), master mix (1X, 12.5µl), and water (0.5µl). *C. jejuni* (ATCC 29411), and *C. coli* (ATCC 43478) were used as a positive control, and nuclease-free water was used as a negative control.

# Quality assurance

# Positive and negative quality control tests are critical for monitoring the overall implementation of the laboratory diagnosis. These help for the development of accurate and reliable data for the study. This can be achieved using positive and negative quality checks. The known quality control organism required for each of the tests in the microbiology laboratory should be conducted accordingly. The following ATCC strains were used for the study of media and reagents according to the manufacturer’s instruction.

# E. coli ATCC 25922, Proteus mirabilis NCTC 10975, *S. enteritidis*ATCC® 14028, Shigella flexneri 12022, *E. coli* O157 ATCC 35150, *C. coli* ATCC 43478, *C. jejuni* ATCC 29411

**Appendix 1. Standard Operating Procedure for Media Preparation, Handling & Disposal**

**Overview**

Growth medium is a solid or liquid substance designed to support the growth of microorganisms or cells. There are different types of media for growing different types of microorganisms or cells. Each medium requires a different set of preparation steps and reagents, and these steps must be conducted properly to be effective. Thus, it is very important to follow certain guidelines during media, and particularly selective supplement preparation to ensure media are properly made. This includes sterility during preparation of media, storage of reagents, and appropriate storage of finished media. Remember: A good medium is KEY in the success of your experiment.

**Tips on handling media**

- Powder to prepare media must be stored appropriately. Read the label of the medium and pay close attention to the storage conditions of each medium (i. e. check if it needs to be stored at 10-30°C, refrigeration temperature, freezer, dry environment etc.).
- Clean lab coats and gloves need to be worn during preparation of media, since contamination of media is a major concern that can produce false results.
- Use plastic weigh boats or paper for weighing media powder.
- Use measuring cylinder to accurately measure the amount of water used to prepare media.
- Use distilled or deionized (DI) water to prepare media. Do not use tap water.
- For mediums which do not require autoclaving (i.e., those that require just boiling), use sterile (autoclaved) distilled water.
- Always prepare larger volumes of media than what is needed (approx. 100 ml extra) to account for the loss of media volume during autoclaving and pouring or aliquoting.
- Autoclave gloves need to be worn when using the autoclave or when handling hot glassware to protect yourself from injury due to contact with hot objects.
- Allow at least a day before the experiment to make your media, unless the medium needs to be prepared on the day of experiments (e.g., MKTTn). Preparation of media takes time, do not rush, and pay attention to details to ensure all the components of the media are properly weighed, diluted, and processed.

The standard autoclave condition (unless otherwise stated) is 121°C at 15 psi for 15 minutes. If large volumes of media per container are autoclaved, the time of autoclaving may need to be increased. 121°C at 15 psi for 20 minutes is also acceptable, however longer autoclaving will result in media browning and changed nutritional composition, which may affect the outcomes of your experiments. To ensure that your media is sterile, use a control medium plate (i.e., incubate a non-inoculated plate or a tube of medium along with your samples to control for sterility).

**General Procedures for Preparing Media**

**Agar Petri Dishes**

1. Calculating Media Amounts:
   1. Calculate the number of media needed:
      1. A standard plate (100 mm x 15 mm) requires $\pm$15 mL of agar.
      2. If 100 plates are needed, then $\pm$1500 mL of agar is needed.
      3. Always make a larger volume of agar than what is needed (approx. 100 ml extra) to account for loss of media during autoclaving and pouring or aliquoting.
   2. Follow the medium label instructions for preparation:
      1. Instructions are typically written to make 1 L of medium or in some cases for 0.5 L medium. If a larger volume is needed, use a ratio equation to calculate the mass required for the desired number of media.

For example, when you need to prepare 400 mL of a medium that requires 30 g of base medium powder for 1 L of medium, calculate the required mass of base medium powder as follows:

**30 g / 1000 mL = X g / 400 mL**

**X = (30 g * 400 mL) / 1000 mL**

**X = 12 g of base medium powder and 400 mL of DI water**

1. Media Making Tips:
2. Always prepare media in a bottle with at least 1/3 empty space (e.g., prepare 700 mL medium in a 1000 mL bottle). This is particularly important when preparing agar media that foam and can overflow during autoclaving. By following these guidelines, you will avoid media loss and damage to your autoclave.
3. Label bottles with the type of media made, your first and last name initials and date prepared. Mark bottles using autoclave tape to verify that the autoclaving was conducted at sufficiently high temperature. For example, a bottle of Tryptic Soy Broth (TSVB) made by Jessie Vipham (JV) on May 15, 2019, should be labeled as follows: **TSB 15/05/2019 JV**
4. Preparing Media:
5. Pour a fraction of the water volume necessary into the bottle before putting in the media powder. This will prevent caking of powders on the bottom of the bottle. After powder is mixed with water, finish pouring the rest of the water into the bottle.
6. Make sure that media powder is fully dispersed in the water. This can be done by manually shaking the bottle, or by inserting a stir bar and mixing the media on a stirring plate. Note: media with high quantity of powder will need to be mixed with a stirring plate; some media may also need to be boiled before autoclaving to prevent caking of the agar on the bottom of the bottle after autoclaving.
7. Boil or autoclave media using the instruction on the media label, to achieve sterility. Do not close the lid on the bottle too tight before autoclaving, to prevent pressure build-up during heating, which can break the bottle. Best practice is to completely close the lid and then open it for half a turn or one full turn. If media is boiled, watch your media constantly and do not let it boil over.
8. While the media is sterilizing, arrange petri dishes on the lab bench top. Depending on the quality of air in your lab, you may need to pour your media in the biosafety cabinet to avoid contamination from air. If the air quality is poor, pouring the media in proximity of the Bunsen burner will help, but will not completely control the contaminants. The best way to test the quality of air in your lab is to leave a nutrient-rich non-selective agar (e.g., BHI, TSA, NA) on the top of the bench for a few hours, with the lid open, and then incubate it at 35 °C for 24 – 48 h and examine the number of colonies.
9. Once the sterilization is completed, allow the media to cool to $\pm$50°C before plating. A good rule of thumb is to plate the media when the media is cool enough to be touched by gloved hands. Avoid shaking the medium prior to pouring into Petri dishes to prevent bubbles on top of the agar, which will make the agar suboptimal for streaking.

***Note:* do not overcool prepared agar as it WILL solidify! Check its temperature by touching the bottle regularly. If you need to keep your agar in the water bath, make sure that every bottle is dried, sprayed with 70% ethanol and wiped before pouring the medium. That will reduce the chance of microbial contamination from water bath.**

1. Pour the desired volume in each petri dish aseptically (approximately 15 mL per standard petri dish). Swirl the plate slightly to ensure the dispersion of agar. If the bubbles are created, you may remove them by flaming the surface of the agar plate, however, that may not be necessary.
2. Allow plates to cool on the countertop. Do not move the plates before they are completely solid, as this will disturb the polymerization of agar. When cooled, invert plates so the lid is on the bottom. It is recommended to dry the plates for approximately 15 – 20 min in a biosafety cabinet before storing them in the fridge to reduce the moisture and prevent condensation during the cold storage.
3. Store plates upside-down (lid side on the bottom) in plastic bags in the refrigerator (-4 °C). Seal and label the bag.

**Broth**

1. Calculate the number of media needed:
   1. Check the protocol to see what volume you need.
   2. Always prepare an extra volume of agar than what is needed (approx. 100 ml extra) to account for loss of media during autoclaving, pouring or aliquoting.
2. Follow the package instructions for preparation:
   1. Instructions are typically written for 1 L or 0.5 L of media. If other volume is needed, use the ratio equation to calculate the grams needed (refer to Agar Petri Dish procedure for sample calculation).
3. Follow steps 3-7 from the Agar Petri Dish procedure.
4. While medium is sterilizing, arrange tubes in racks on the countertop or in the biosafety cabinet, as needed.
5. Once sterilization is completed, allow the media to cool.
6. Aseptically aliquot the desired volume of medium in each tube using a pipette or a dispenser. Place caps onto the tubes.
7. Store broths in the refrigerator to prolong the shelf life.

**Storage**

- Prepared media (agar and broths) that are not used immediately must be stored in the refrigerator. Antibiotic stock must be stored in the freezer.
- For quality purposes, do not use agars and broths that are stored more than three weeks in the refrigerator.

**Identifying contamination on prepared media**

It is best practice to inspect prepared media for contamination before using. Media can be contaminated due to several reasons such as a breach in sterility during plating/dispensing, improper storage of media etc. Following are signs that indicate your media are contaminated:

- Fungal growth on agar: fuzzy, filamentous or hairlike growths and visible.
- Bacterial growth on agar: presence of bacterial colonies on an un-inoculated agar.
- Color change of media (both agar and broth).
- Turbid broth: cloudy broth, substance floating in the broth.

****Contaminated media must not be used to conduct experiments and must be properly discarded (i.e., needs to be autoclaved before discarding). If you believe that a batch of plates may have been contaminated, place them in a 37°C incubator or a warm location overnight, then inspect for signs of contamination as described above. ****

**Disposal of inoculated or contaminated media**

Inoculated or contaminated media must be sterilized before they are disposed of or cleaned. These are the steps used to sterilize and dispose inoculated media in plastic petri dish and tubes:

1. Secure Petri dish or plastic tubes in a plastic autoclave bag. It is a best practice to “double bag” (i.e. put the autoclaved bag filled with media inside another empty autoclave bag) to avoid leakage or place the bag in a secondary container (a plastic container that will contain the liquid if your bag breaks).
2. Seal the bag using autoclave tape.
3. Sterilize at 121°C at 15 psi for 30 minutes.
4. Let cool and dispose the autoclave bag in a trash bin. Never pour liquid agar into the sink, as it will clog the drain.

**Disposal of inoculated media in glassware**:

1. Secure glass tubes in metal racks.
2. Mark glassware or racks with autoclave tape.
3. Ensure that all the lids are slightly open to allow for pressure release.
4. Sterilize at 121°C at 15 psi for 30 minutes.
5. Drain contents into the sink under running tap water. Wash glassware using detergent and rinse using distilled water. Never pour liquid agar into the sink, as it will clog the drain.
6. Let dry.

***Salmonella* spp. Media Preparation Guide**

**Selenite F broth**

Principle: **Selenite F Broth** is the medium used for the selective enrichment of Salmonella spp from both clinical and food samples. It is a buffered Lactose Peptone Broth to which Sodium Biselenite is added as the selective agent. **Casein enzymic hydrolysate** provides nitrogenous substances, carbon compounds and vitamin sources. **Lactose** is a carbohydrate source which maintains the pH of medium. **Selenite** is reduced by bacterial growth and alkali is produced. An increase in pH lessens the toxicity of the selenite and results in overgrowth of other bacteria. The acid produced by bacteria due to lactose fermentation serves to maintain a neutral pH. **Sodium phosphate** maintains a stable pH and lessens the toxicity of selenite. **Sodium selenite** inhibits many species of gram-positive and gram-negative bacteria including enterococci and coliforms. Do not incubate the broth longer than 24 hours as inhibitory effect of selenite decreases after 6 – 12 hours of incubation. It is used as a selective enrichment for the cultivation of Salmonella spp, an enrichment medium for the isolation of some species of Shigella, and the broth is also recommended for the transport of strains of Vibrio cholerae, because these organisms can survive 2 to 5 days in Sodium Selenite Broth.

### Preparation of Selenite F Broth

1. Add 4.0 gm sodium selenite powder to distilled/deionized water (500ml).
2. Add the remaining 19.0 gm powder to above solution and distilled/ deionized water and bring the total volume to 1.0 liter.
3. Gently heat and bring to boiling.
4. Dispense into sterile test tubes (10ml each)
5. Sterilize in boiling water bath or at 0 psi pressure at 100°C for 10 minutes.
6. **DO NOT AUTOCLAVE.**
7. Cool to room temperature prior to use.

**Xylose Lysine Deoxycholate (XLD)**

Type: Agar, Supplements: None

Suspend 56.68 grams of base media powder in 1000 ml sterile distilled water. Heat with frequent agitation until the medium boil. BE CAREFUL. Once the medium begins to boil, the bubbles will move up the flask very quickly, and the medium will boil over if not removed from heat. The bubbles should reach the upper part of the flask before quickly removing the medium from heat. If the medium appears grainy and translucent, the flask should be heated again. If the medium appears transparent and does not contain grainy agar crystals, the medium can begin cooling. DO NOT AUTOCLAVE OR OVERHEAT. Transfer immediately to a water bath at 50°C, if available (if not, cool down on a bench top). After cooling, pour into sterile Petri plates.

**Note:** It is recommended not to prepare large volumes that will require prolonged heating at once. Plan the preparation of this agar well ahead of time. There are many things that can go wrong with this agar and the instructions need to be followed exactly to achieve the desired quality of the medium. For example, if not heated as instructed, the solids in the agar will not completely dissolve, the agar will not solidify as expected and may fall out of the petri dishes when incubated.

**MacConkey Agar**

Suspend 49.53 grams of dehydrated medium in 1000 ml purified/distilled water. Heat to boiling to dissolve the medium completely. Sterilize by autoclaving at 15 lbs pressure (121°C) for 15 minutes.  Cool to 45-50°C. Mix well before pouring into sterile Petri plates.

**STEC Media Preparation Guide**

**CHROMagarTM STEC**

Step1. Preparation of the base CHROMagarTM STEC base

- Disperse slowly 30.8 g of powder base in 1 L of purified water.
- Stir until agar is well thickened.
- Heat and bring to boil (100 °C) while swirling or stirring regularly.
  - DO NOT HEAT TO MORE THAN 100 °C.
  - DO NOT AUTOCLAVE AT 121 °C.
  - Warning 1: If using an autoclave, do so without pressure.
  - Advice 1: For the 100 °C heating step, mixture may also be brought to a boil in a microwave oven: after initial boiling, remove from oven, str gently, then return to oven for short, repeated bursts of heating until complete fusion of the agar grains has taken place (large bubbles replacing foam).

• Cool in a water bath to 45-50 °C. Swirl or str gently to homogenize.

Step2. Preparation of the Supplement (S) and Mix of the prepared mix.

- Aseptically rehydrate ONE vial with 10 mL of sterile water. Use 1 vial rehydrated supplement per 1 liter of media. 5 liters, needs 5 vial supplements.
- Swirl well until complete dissolution.
- Add this rehydrated solution to the CHROMagarTM STEC base cooled at 45-50 °C.
- Swirl gently to homogenize.

Step 3. Pouring plate

- Pour into sterile Petri dishes.
- Let it solidify and dry

Storage:

- Store in the dark before use.
- Prepared media plates can be kept for one day at room temperature.

Advice 2: Plates can be stored for up to one month under refrigeration (2/8 °C) if properly prepared and protected from light and dehydration.

Advice 3: If not fully used, rehydrated CHROMagarTM STEC supplement can be stored up to 2 months at 2/8 °C.

**Bacterial Isolates Storage media Preparation**

**Trypticase Soy Broth plus 20% glycerol**

Trypticase soy broth (BD)-------------------- 30.0g

ETF (endotoxin-free) H_2_O-------------------- 800.0 ml

Glycerol (Baker) ------------------------------ 200.0 ml

1. Add water to dehydrated trypicase soy broth base; heat to dissolve the medium completely.
2. Add glycerol and dispense in 1.0ml aliquots in 12mm x 75 mm sterile, sealed tubes.

Sterilize by autoclaving for 15min at 121^o^c.

1. Store at 4^o^C or -20^o^C (long term) or at room temp until used. Storage conditions: Refrigerated, 4-10^o^C (long-term) or room temp. Shelf life: 1 year
