# Supplementary material for: Prevalence of Shiga toxin-producing Escherichia coli, Salmonella, and Campylobacter species among diarrheal patients from three major hospitals in Ethiopia
Source: PLOS Glob Public Health. 2025 Apr 21;5(4):e0004407. doi: 10.1371/journal.pgph.0004407 (PMC12011234; doi:10.1371/journal.pgph.0004407)
Supplement: S1 Table — (DOCX) [file pgph.0004407.s004.docx]

**S1 Table: Prevalence of selected demographics and Clinical Information for patients with Confirmed Pathogens of interest.**

| **Characteristics** | **Categories** | **Total** | **NTS** | | **STEC (*stx* only)** | | **STEC (*stx* + *eae*)** | | **CAMPY** | |
| --- | --- | --- | --- | --- | --- | --- | --- | --- | --- | --- |
|  |  |  | **n (%)** | **Prevalence (95%CI)** | **n (%)** | **Prevalence (95%CI)** | **n (%)** | **Prevalence (95%CI)** | **n (%)** | **Prevalence (95%CI)** |
| **Study sites** | Addis Ababa | 792 | 2 (0.30) | 0.25 (0.07,0.92) | 118 (14.90) | 14.90 (12.59,17.55) | 26 (3.28) | 3.28 (2.24,4.77) | 21 (2.65) | 2.65 (1.74,4.02) |
|  | Gondar | 767 | 6 (0.80) | 0.78 (0.36,1.70) | 58 (7.56) | 7.56 (5.90,9.65) | 22 (2.87) | 2.87 (1.90,4.30) | 36 (4.69) | 4.69 (3.41,6.43) |
|  | Harar | 772 | 22 (2.80) | 2.85 (1.89,4.28) | 117 (15.15) | 15.16 (12.80,17.86) | 32 (4.15) | 4.15 (2.95,5.79) | 47 (6.10) | 6.10 (4.61,8.00) |
|  | Overall | 2331 | 30 (1.29) | 1.29 (0.90, 1.83) | 293 (12.56) | 12.56 (11.29, 13.98) | 80 (3.43) | 3.43 (2.77, 4.25) | 104 (4.46) | 4.46 (3.70, 5.38) |
| **Age groups** | <5 | 328 | 6 (20.00) | 1.83 (0.84,3.93) | 41 (13.99) | 12.50 (9.35,16.52) | 8 (10.00) | 2.44 (1.24,4.74) | 30 (28.85) | 9.15 (6.48,12.76) |
|  | 5-14 | 228 | 3 (10.00) | 1.32 (0.45,3.80) | 23 (7.85) | 10.09 (6.82,14.68) | 7 (8.75) | 3.07 (1.49,6.20) | 28 (26.92) | 12.28 (8.63,17.18) |
|  | 15-19 | 107 | 1 (3.00) | 0.93 (0.17,5.10) | 15 (5.12) | 14.02 (8.68,21.85) | 3 (0.38) | 2.80 (0.96,7.92) | 1 (0.96) | 0.93 (0.17,5.10) |
|  | 20-29 | 548 | 4 (13.30) | 0.73 (0.28,1.86) | 76 (25.94) | 13.87 (11.23,17.01) | 25 (31.25) | 4.56 (3.11,6.65) | 14 (13.46) | 2.55 (1.53,4.24) |
|  | 30-39 | 412 | 8 (27.00) | 1.94 (0.99,3.78) | 40 (13.65) | 9.71 (7.21,12.95) | 11 (13.75) | 2.67 (1.50,4.72) | 11 (10.57) | 2.67 (1.50,4.72) |
|  | 40-49 | 297 | 3 (10.00) | 1.01 (0.34,2.93) | 44 (15.02) | 14.77 (11.19,19.24) | 7 (8.75) | 2.36 (1.15,4.78) | 10 (9.61) | 3.37 (1.84,6.09) |
|  | 50+ | 411 | 5 (16.70) | 1.22 (0.52,2.82) | 54 (18.43) | 13.17 (10.24,16.79) | 19 (23.75) | 4.62 (2.98,7.11) | 10 (9.61) | 2.43 (1.33,4.42) |
| **Sex** | Male | 1277 | 17 (56.70) | 1.33 (0.83,2.12) | 160 (54.61) | 12.53 (10.83,14.46) | 45 (56.25) | 3.52 (2.64,4.68) | 65 (62.50) | 5.09 (4.01,6.43) |
|  | Female | 1054 | 13 (43.30) | 1.23 (0.72,2.10) | 133 (45.39) | 12.62 (10.75,14.76) | 35 (43.75) | 3.32 (2.40,4.58) | 39 (37.51) | 3.70 (2.72,5.02) |
| **Residence Urbanicity** | Urban | 1961 | 21 (70.00) | 1.07 (0.70,1.63) | 255 (87.03) | 13.00 (11.59,14.56) | 71 (88.75) | 3.62 (2.88,4.54) | 79 (75.96) | 4.03 (3.24,4.99) |
|  | Rural | 370 | 9 (30.00) | 2.43 (1.28,4.56) | 38 (12.97) | 10.27 (7.57,13.78) | 9 (11.25) | 2.43 (1.28,4.56) | 25 (24.04) | 6.77 (4.62,9.78) |
| **Season** | Dry Season | 1141 | 16 (53.30) | 1.40 (0.86,2.27) | 160 (54.61) | 14.02 (12.13,16.16) | 47(58.75) | 4.12(3.11,5.43) | 48 (46.15) | 4.21 (3.19,5.53) |
|  | Short Rains | 526 | 5 (16.70) | 0.95 (0.41,2.21) | 38 (12.97) | 7.22 (5.31,9.76) | 13 (16.25) | 2.47(1.45,4.18) | 19 (18.27) | 3.61 (2.32,5.57) |
|  | Long Rains | 664 | 9 (30.00) | 1.36 (0.71,2.56) | 95 (32.42) | 14.31 (11.85,17.18) | 20 (25.00) | 3.01 (1.96,4.61) | 37 (35.58) | 5.57 (4.07,7.59) |
| **Types of Diarrhea** | Watery diarrhea | 1622 | 25 (83.30) | 0.02 (0.01,0.02) | 202 (68.94) | 12.45 (10.94,14.15) | 62 (77.51) | 0.04 (0.03, 0.05) | 89 (85.60) | 0.06 (0.05,0.07) |
|  | Bloody diarrhea | 287 | 4 (13.30) | 0.01 (0.01,0.04) | 34 (11.60) | 11.85 (8.60,16.10) | 12 (15.00) | 0.04 (0.02,0.07) | 11 (10.60) | 0.04 (0.02,0.07) |
|  | Mucoid stool | 430 | 2 (6.70) | 0.01 (0.00,0.02) | 46 (15.70) | 10.70 (8.12,13.97) | 17 (21.25) | 0.04 (0.03, 0.06) | 24 (23.10) | 0.06 (0.04,0.08) |
| **Signs and Symptoms** | Abdominal cramps | 1357 | 22 (73.30) | 0.02 (0.01,0.02) | 188 (64.16) | 13.85 (12.12,15.79) | 56 (70.00) | 0.04 (0.03,0.05) | 63 (60.61) | 0.05 (0.04,0.06) |
|  | Undefined abdominal pain | 1534 | 23 (76.70) | 0.02 (0.01,0.02) | 216 (73.72) | 14.08 (12.43,15.91) | 59 (73.75) | 0.04 (0.03, 0.05) | 72 (69.22) | 0.05 (0.04,0.06) |
|  | Fever | 812 | 16 (53.30) | 0.02 (0.01,0.03) | 121 (41.30) | 14.90 (12.62,17.52) | 37 (46.25) | 0.05 (0.03, 0.06) | 56 (53.8) | 0.07 (0.05,0.09) |
|  | Bloating | 226 | 0 (0.00) | 0 (0.00,0.00) | 33 (11.26) | 14.60 (10.59,19.79) | 9 (11.25) | 0.04 (0.02,0.07) | 8 (7.70) | 0.04 (0.02,0.07) |
|  | Nausea | 390 | 4 (13.30) | 0.01 (0.00,0.03) | 50 (17.06) | 12.82 (9.86,16.50) | 11 (13.75) | 0.03 (0.02, 0.05) | 22 (21.20) | 0.06 (0.04,0.08) |
|  | Vomiting | 444 | 8 (26.67) | 0.02 (0.01,0.02) | 72 (24.57) | 16.22 (13.08,19.93) | 19 (23.75) | 0.04 (0.03, 0.07) | 33 (31.71) | 0.07 (0.05,0.10) |
|  | None | 25 | 0 (0.00) | 0(0.00,0.00) | 4 (1.37) | 16.00 (6.40,34.65) | 1 (1.25) | 0.05 (0.01, 0.23) | 3 (2.90) | 0.14 (0.05,0.35) |
